# Supplementary material for: Risk factors for hospitalisation in Welsh infants with a congenital anomaly
Source: BMJ Paediatr Open. 2022 Feb 11;6(1):e001238. doi: 10.1136/bmjpo-2021-001238 (PMC8845320; doi:10.1136/bmjpo-2021-001238)
Supplement: Supplementary data [file bmjpo-2021-001238supp001.pdf]

Supplementary Table 1: Inclusion of anomaly and rare disease of congenital origin subgroups.

Subgroup	Classification of anomaly and rare disease of congenital origin included
All anomalies; all isolated anomalies; and all multiple anomalies	• Nervous system;<br>• Eye, ear, face and neck;<br>• Cardiovascular system;<br>• Respiratory and clefts;<br>• Abdominal wall defects and diaphragmatic hernia;<br>• Upper and lower gastrointestinal;<br>• Genitourinary;<br>• Limb and skeletal;<br>• Blood disorders;<br>• Endocrine and Metabolic;<br>• Neoplasm;<br>• Syndromes and congenital malformation syndromes;<br>• Maternal Infection leading to malformations.
Cardiovascular anomalies	• Congenital heart defects and diseases of the circulatory system.

Ho PSY, *et al. BMJ Paediatrics Open* 2022; 6:e001238. doi: 10.1136/bmjpo-2021-001238

Supplementary Table 2: Year of birth added as a covariate in the final model as a sensitivity analysis.

	2+ admissions				1 admission			
	Adjusted OR				Adjusted OR			
Factors	All	Isolated	Multiple	Cardiovascular	All	Isolated	Multiple	Cardiovascular
Maternal age at delivery, years								
≤ 24	1.18 (1.06-1.30)		1.22 (1.06-1.41)		1.00 (0.89-1.12)		1.04 (0.88-1.24)	
25 – 29	1 (reference)		1 (reference)		1 (reference)		1 (reference)	
30 – 34	0.99 (0.89-1.10)		0.94 (0.81-1.09)		0.94 (0.83-1.06)		0.90 (0.75-1.07)	
≥ 35	0.93 (0.82-1.04)		0.54 (0.47-0.63)		0.95 (0.84-1.09)		0.58 (0.48-0.69)	
Maternal smoking								
Smoker	1.20 (1.10-1.31)	1.26 (1.13-1.41)			1.06 (0.96-1.17)	1.04 (0.92-1.18)		
Non/Ex smoker	1 (reference)	1 (reference)			1 (reference)	1 (reference)		
Infant sex[1]								
Male	1 (reference)	1 (reference)	1 (reference)	1 (reference)	1 (reference)	1 (reference)	1 (reference)	1 (reference)
Female	0.84 (0.78-0.91)	0.82 (0.74-0.90)	0.90 (0.81-1.00)	0.75 (0.62-0.91)	0.95 (0.87-1.04)	1.01 (0.91-1.12)	0.87 (0.77-0.99)	0.95 (0.78-1.15)
Gestational age[1], week								
< 37	2.52 (2.25-2.83)	2.83 (2.41-3.31)	2.19 (1.90-2.53)	3.74 (2.84-4.91)	1.68 (1.47-1.92)	1.85 (1.55-2.21)	1.50 (1.26-1.79)	1.97 (1.45-2.64)
≥ 37	1 (reference)	1 (reference)	1 (reference)	1 (reference)	1 (reference)	1 (reference)	1 (reference)	1 (reference)
CHD Disease Severity[1]								
Less severity	N/A	N/A	N/A	1 (reference)	N/A	N/A	N/A	1 (reference)
Moderate severity				6.31 (4.51-8.83)				1.20 (0.78-1.84)
Most severe				2.76 (1.60-4.77)				0.39 (0.16-0.98)
For each year increment between 1999 and 2016.	1.00 (0.99-1.01)	1.00 (0.99-1.01)	1.00 (0.99-1.01)	0.99 (0.97-1.01)	0.99 (0.98-1.00)	0.99 (0.97-1.00)	1.00 (0.98-1.01)	0.98 (0.96-1.00)

[1]*Variable identified for inclusion a priori. N/A = not applicable.*

Supplementary Table 3: Factors associated with cause-specific admissions among infants with any anomaly or rare disease of congenital origin.

	Anomaly-specific admissions, no. of infants (%)	Other-cause admissions, no. of infants (%)	Unadjusted OR (95% CI)	P-value[1]
Socio-demographic factors				
Townsend quintile				
1 (least deprived)	772 (15.4)	1,709 (14.9)	1 (reference)	0.253
2	767 (15.3)	1,780 (15.6)	0.95 (0.85-1.08)	
3	932 (18.6)	2,042 (17.9)	1.01 (0.90-1.13)	
4	1,093 (21.8)	2,432 (21.3)	0.99 (0.89-1.11)	
5 (most deprived)	1,439 (28.8)	3,472 (30.4)	0.92 (0.83-1.02)	
Maternal ethnicity				
White	3,101 (94.2)	6,948 (94.2)	1 (reference)	0.978
Other	191 (5.8)	429 (5.8)	1.00 (0.84-1.19)	
Maternal age at birth, years *(n = 29,400)*				
≤ 24	1,570 (31.3)	3,680 (32.1)	0.97 (0.89-1.06)	0.712
25 – 29	1,363 (27.1)	3,103 (27.1)	1 (reference)	
30 – 34	1,251 (24.9)	2,782 (24.3)	1.02 (0.93-1.22)	
≥ 35	831 (16.6)	1,891 (16.5)	1.00 (0.90-1.11)	
Maternal factors				
Parity				
Nulliparous	2,101 (41.9)	4,782 (41.7)	1 (reference)	0.856
≥ 1	2,914 (58.1)	6,674 (58.3)	0.99 (0.93-1.06)	
Multiple fetus				
Yes	153 (3.1)	427 (3.7)	0.81 (0.67-0.98)	0.028
No	4,862 (96.9)	11,029 (96.3)	1 (reference)	
Maternal smoking				
Smoker	946 (28.0)	2,218 (30.1)	0.90 (0.83-0.99)	0.027
Non/ Ex smoker	2,430 (72.0)	5,147 (69.9)	1 (reference)	
Anomalies in previous pregnancies				
Yes	374 (10.4)	1,028 (12.8)	1.27 (1.12-1.44)	<0.001
No	3,231 (89.6)	6,981 (87.2)	1 (reference)	
Infant factors				
Infant sex				
Male	3,013 (60.1)	6,964 (60.8)	1 (reference)	0.405
Female	1,999 (39.9)	4,489 (39.2)	1.03 (0.96-1.10)	
Birthweight, grams				
< 2500	598 (11.9)	2,448 (21.4)	0.50 (0.45-0.55)	<0.0001
≥ 2500	4,417 (88.0)	9,008 (78.6)	1 (reference)	
Gestational age, week				
$< 37^{+0}$	577 (11.5)	2,470 (21.6)	0.47 (0.43-0.52)	<0.0001
$\geq 37^{+0}$	4,438 (88.5)	8,986 (78.4)	1 (reference)	

[1]P < 0.1 was considered as statistically significant. Missing data are not shown due to risk of statistical disclosure.

Supplementary Table 4: Factors associated with cause-specific admissions among infants with any anomaly or rare disease of congenital origin: multivariable analysis.

Risk factors	Crude OR (95% CI)	P-value[1]	aOR[2] (95% CI)	P-value[3]
Anomalies in previous pregnancies				
Yes	1.27 (1.12-1.44)	<0.001	1.26 (1.11-1.43)	<0.001
No	1 (reference)		1 (reference)	
Gestational age, week				
< 37 (preterm)	0.47 (0.43-0.52)	<0.0001	0.47 (0.42-0.52)	<0.0001
≥ 37 (term)	1 (reference)		1 (reference)	

[1]P-value of the univariable analysis < 0.1 was considered as statistically significant. [2]Mutually adjusted odds ratios for other factors in the table. [3]P-value of the multivariable analysis < 0.05 was considered as statistically significant.

Ho PSY, *et al. BMJ Paediatrics Open* 2022; 6:e001238. doi: 10.1136/bmjpo-2021-001238
