# Supplementary material for: Risk factors for hospitalisation in Welsh infants with a congenital anomaly
Source: BMJ Paediatr Open. 2022 Feb 11;6(1):e001238. doi: 10.1136/bmjpo-2021-001238 (PMC8845320; doi:10.1136/bmjpo-2021-001238)
Supplement: Author's manuscript [file bmjpo-2021-001238.draft_revisions.pdf]

BMJ Paediatrics Open

BMJ Paediatrics Open is committed to open peer review. As part of this commitment we make the peer review history of every article we publish publicly available.

When an article is published we post the peer reviewers' comments and the authors' responses online. We also post the versions of the paper that were used during peer review. These are the versions that the peer review comments apply to.

The versions of the paper that follow are the versions that were submitted during the peer review process. They are not the versions of record or the final published versions. They should not be cited or distributed as the published version of this manuscript.

BMJ Paediatrics Open is an open access journal and the full, final, typeset and author-corrected version of record of the manuscript is available on our site with no access controls, subscription charges or pay-per-view fees (http://bmjpaedsopen.bmj.com).

If you have any questions on BMJ Paediatrics Open's open peer review process please email info.bmjpo@bmj.com

BMJ Paediatrics Open

RISK FACTORS FOR HOSPITALISATION IN INFANTS WITH A CONGENITAL ANOMALY.

Journal:	*BMJ Paediatrics Open*
Manuscript ID	bmjpo-2021-001238
Article Type:	Original research
Date Submitted by the Author:	28-Jul-2021
Complete List of Authors:	Ho, Peter ; University of Oxford, Nuffield Department of Population Health<br>Quigley, Maria; University of Oxford, National Perinatal Epidemiology Unit, Nuffield Department of Population Health; University of Oxford, National Institute for Health Research (NIHR) Policy Research Unit-Maternal Health and Care, National Perinatal Epidemiology Unit, Nuffield Department of Population Health<br>Tucker, David; Public Health Wales, Congenital Anomaly Register and Information Service (CARIS) for Wales<br>Kurinczuk, Jenny; University of Oxford, National Perinatal Epidemiology Unit, Nuffield Department of Population Health; University of Oxford, National Institute for Health Research (NIHR) Policy Research Unit-Maternal Health and Care, National Perinatal Epidemiology Unit, Nuffield Department of Population Health
Keywords:	Epidemiology, Neonatology, Health services research

SCHOLARONE™
Manuscripts

I, the Submitting Author has the right to grant and does grant on behalf of all authors of the Work (as defined in the below author licence), an exclusive licence and/or a non-exclusive licence for contributions from authors who are: i) UK Crown employees; ii) where BMJ has agreed a CC-BY licence shall apply, and/or iii) in accordance with the terms applicable for US Federal Government officers or employees acting as part of their official duties; on a worldwide, perpetual, irrevocable, royalty-free basis to BMJ Publishing Group Ltd ("BMJ") its licensees and where the relevant Journal is co-owned by BMJ to the co-owners of the Journal, to publish the Work in this journal and any other BMJ products and to exploit all rights, as set out in our licence.

The Submitting Author accepts and understands that any supply made under these terms is made by BMJ to the Submitting Author unless you are acting as an employee on behalf of your employer or a postgraduate student of an affiliated institution which is paying any applicable article publishing charge ("APC") for Open Access articles. Where the Submitting Author wishes to make the Work available on an Open Access basis (and intends to pay the relevant APC), the terms of reuse of such Open Access shall be governed by a Creative Commons licence – details of these licences and which Creative Commons licence will apply to this Work are set out in our licence referred to above.

Other than as permitted in any relevant BMJ Author's Self Archiving Policies, I confirm this Work has not been accepted for publication elsewhere, is not being considered for publication elsewhere and does not duplicate material already published. I confirm all authors consent to publication of this Work and authorise the granting of this licence.

Confidential: For Review Only

RISK FACTORS FOR HOSPITALISATION IN INFANTS WITH A CONGENITAL ANOMALY.

Peter Ho[1], Maria A Quigley[1,2], David Tucker[3], Jennifer J Kurinczuk[1,2]

[1]National Perinatal Epidemiology Unit, Nuffield Department of Population Health, University of Oxford.

[2]National Institute for Health Research (NIHR) Policy Research Unit- Maternal Health and Care, National Perinatal Epidemiology Unit, Nuffield Department of Population Health, University of Oxford.

[3]Congenital Anomaly Register and Information Service (CARIS) in Wales.

Correspondence author: Dr. Peter Ho, National Perinatal Epidemiology Unit, Nuffield Department of Population Health, University of Oxford, Old Road Campus, Headington, Oxford OX3 7LF, UK. Email: Peter.ho@gtc.ox.ac.uk, Peterhosy@hotmail.com Tel: +44 7905497825 Fax: +44 1865 289701

2500 words (excluding abstract and references)

Confidential: For Review Only

Abstract

Objectives: To investigate risk factor associated with hospitalisation of infants with a congenital anomaly in Wales, UK.

Design: A population-based cohort study.

Setting: Data from the Welsh Congenital Anomaly Register and Information Service (CARIS) linked to the Patient Episode Database for Wales (PEDW) and livebirths and deaths from the Office for National Statistics (ONS).

Patients: All livebirths between 1999 and 2015 with a diagnosis of a congenital anomaly, which was defined as a structural, metabolic, endocrine, or genetic defect, as well as rare diseases of hereditary origin.

Main outcome measures: Adjusted relative risk ratios (aRRR) associated with 1 or 2+ hospital admissions in infancy versus no admissions were estimated for socio-demographic, maternal and infant factors using multinomial logistic regression for the subgroups of all, isolated, multiple and cardiovascular anomalies.

Results: 25,523 infants affected by congenital anomalies experienced a total of 50,705 admissions in infancy. Risk factors for ≥2 admissions were younger maternal age ≤24 years (aRRR: 1.17; 95% CI: 1.06-1.30), maternal smoking (aRRR: 1.20; 1.10-1.31), preterm birth (aRRR: 2.52; 2.25-2.82) and moderately severe congenital heart defects (aRRR: 6.25; 4.47-8.74). Girls had an overall decreased risk of 2+ admissions (aRRR: 0.84; 0.78-0.91). Preterm birth was a significant risk factor for admissions in

Confidential: For Review Only

all anomaly subgroups but the effect of the other characteristics varied according to anomaly subgroup.

Conclusions: Over two thirds of infants with an anomaly are admitted to hospital during infancy. Our findings identified socio-demographic and clinical characteristics contributing to an increased risk of hospitalisation of infants with congenital anomalies.

Confidential: For Review Only

Introduction

Congenital anomalies are a leading cause of infant morbidity and hospitalisation [1]. Infants who are born with a major congenital anomaly such as a congenital heart defect (CHD), Down syndrome and congenital diaphragmatic hernia often experience anomaly-related sequelae and complications, and require more frequent hospitalisation compared to children without an anomaly.

There is some evidence that infant characteristics can influence hospitalisation of children affected by specific anomalies. For example, previous studies have shown that preterm birth, low birthweight, disease severity and co-morbidity are significantly associated with an increased risk of excess hospitalisation of infants with spina bifida, choanal atresia, congenital heart defects, orofacial clefts, congenital diaphragmatic hernia, biliary atresia, gastroschisis and Down syndrome [2-8]; while the effects of socio-demographic and maternal are less well established. Further research on risk factors for hospitalisation, particularly those associated with a wider range of congenital anomalies are needed to inform planning of healthcare and social interventions aimed at reducing infant morbidity and hospitalisation.

We aimed to investigate risk factors for hospitalisation in the first year after birth among infants with congenital anomalies.

Confidential: For Review Only

Methods

Study design

A national population-based cohort study was established using registry data from the Congenital Anomaly Register and Information Service (CARIS) for Wales, linked to Patient Episode Database for Wales (PEDW), and births and deaths registration data from the Office for National Statistics (ONS) [9-11], and de-identified for analysis.

Study population

The inclusion criteria for this study were:

1. All livebirths between 1999 and 2015 with birthweight ≥500g, gestational age ≥22^{+0} weeks, with a confirmed or probable diagnosis of a congenital anomaly reported to CARIS. CARIS defines congenital anomalies as structural, metabolic, endocrine, or genetic defects, as well as rare diseases of hereditary origin present in the child or fetus at the end of pregnancy, even if not detected until after birth [9]. Therefore, the 10th revision of International Classification of Diseases and Related Health Problems (ICD-10) 'Q', 'P35' and 'P37" codes, as well as other non 'Q' ICD-10 codes of congenital anomalies and rare diseases were also included in this study.
2. All hospital admissions (i.e. emergency, elective and transfer admissions) of infants with a congenital anomaly. An admission is defined as a hospital stay of a patient using a bed on premises controlled by the one health care provider [12].

Confidential: For Review Only

An admission is ended by a discharge, transfer to another NHS provider or death. All infants who were admitted from birth due to medical reasons were included in the analysis.

Exclusion criteria

1. Admissions solely for the birth of babies (i.e. birth hospitalisation) were excluded.

Categorisation of anomaly

Congenital anomalies were categorised according to the European Surveillance of Congenital Anomalies (EUROCAT) subgroup classification [13]; other rare diseases which are not included in the EUROCAT subgroup classification were categorised according to the ICD chapter headings [14] (Supplementary Table 1).

Infants were considered as having an isolated congenital anomaly (or disease) if a single anomaly (or disease) was diagnosed and reported to CARIS. Infants were considered as having multiple anomalies (or diseases) if more than one anomaly (or diseases) was diagnosed, either within the same body system or involving different body systems. Infants who were diagnosed with a syndrome involving more than one anomaly (or disease) were considered as having multiple anomalies (or diseases).

Confidential: For Review Only

Outcome

The outcome was total hospital admissions for any cause in infancy (period from birth up to the first birthday) and infants were categorised as having 0, 1 or 2+ admissions.

Statistical analysis

Multinomial logistic regression was conducted to estimate the adjusted relative risk ratios (aRRR) of hospital admission and the corresponding 95% confidence interval (CI) according to the categories of socio-demographic, maternal and infant factors (Table 1), comparing infants who had one or 2+ admissions versus those who had no admission (the comparison group). Any hospital transfers were treated as separate admissions in order to assess the full burden and utilisation of hospitalisation.

Variables included *a priori* in the multivariable models were infant sex, gestational age at birth and disease severity for cardiovascular anomalies as these are known determinants of infant morbidity and hospitalisation [5, 15-18]. Variables that were associated with hospitalisation in univariable analysis ($p < 0.1$) were also explored in multivariable models. Variables were removed from the multivariable model if no category of the variable statistically improved the fit of the data (i.e. $p < 0.05$). As birthweight and gestational age at birth were highly collinear (correlation coefficient $r = 0.75$), only gestational age at birth was included in the final model due to its greater clinical utility. All multivariable analysis was carried out separately for the following subgroups: infants with any anomaly; infants with isolated anomalies; infants with multiple anomalies; and infants with cardiovascular anomalies.

Confidential: For Review Only

A further subgroup analysis was conducted for those babies who had at least one admission to investigate whether risk factors were different between infants with anomaly-specific admissions and those whose admissions were for 'other-causes'. Here, anomaly-specific admissions were defined as hospitalisation of infants due to 'congenital anomalies, deformations, and chromosomal abnormalities' (ICD-10 codes: Q00-Q99/ chapter XVII) as the primary reason for admission. Crude odds ratios with corresponding 95% CIs were estimated for each explanatory variable comparing these two groups of infants. Significant variables from the univariable analysis ($p < 0.1$) were included in the multivariable analysis. $P < 0.05$ was considered as statistically significant in all final models.

Missing data for the majority of variables were few and they are not shown due to the risk of statistical disclosure. All analyses were performed using complete case analysis. All statistical analyses were performed using Stata 13 [19].

Research ethics and statistical disclosure

The secondary anonymised health data for this study was provided by the Secure Anonymised Information Linkage (SAIL) databank in Wales. This study was approved by the SAIL Information Governance Review Panel (IGRP) and did not require separate research ethics committee review. All statistical disclosure control and checks were strictly followed according to the SAIL Databank and ONS guidelines, to ensure that individual infants were not deductively identifiable.

Confidential: For Review Only

Patient and public involvement

All proposals to use data within the SAIL Databank are subject to review by an independent Information Governance Review Panel (IGRP) for privacy risk, data governance and public benefit assessment. The IGRP is made up of a range of independent experts as well as members of the public.

Results

Between 1999 and 2016, 25,523 babies were born alive and affected by congenital anomalies or rare disease of congenital origin, and required a total of 50,705 admissions in infancy. Overall, approximately a quarter of infants had only one admission, over 40% had two or more admissions and more than one third had no admission in infancy. Among infants with isolated anomalies, the largest number of admissions occurred in infants with a cardiovascular anomaly (8% of all admissions of babies with an anomaly).

Table 1 shows that infants who had two or more admissions compared with those who had one or no admission were more likely to live in a deprived area (30.6% versus 28.6% or 26.7%), to have mothers who were younger (33.3% versus 29.3% or 28.2%), nulliparous (45.2% versus 43.6% or 43.3%), and smokers (30.7% versus 27.1% or 25.5%), to be boys (61.2% versus 59.6% or 59.9%), born preterm (21.3% versus 14.0% or 9.1%), to have a low birthweight (21.5% versus 13.5% or 10.1%), or to be one of a multiple pregnancy (3.9% versus 2.9% or 2.4%).

Confidential: For Review Only

In the univariable analysis, social deprivation, maternal age, parity, maternal smoking, and preterm birth were significantly associated with an increased or decreased risk for repeated admissions for ‘all’, ‘all isolated’ and ‘all multiple’ anomaly subgroups (Table 2). Except for social deprivation and maternal smoking, the same variables (i.e. maternal age and preterm births) which were associated with an increased risk for repeated admissions were also consistently associated with an increased or decreased risk of single admissions of infants for these anomaly subgroups.

Tables 3 and 4 shows the adjusted RRRs for variables significantly associated with ($p < 0.05$) the number of admissions in infancy by different anomaly or rare disease of congenital origin subgroups. Preterm birth was consistently the strongest risk factor associated with increased 1 and 2+ admissions of infants across all anomalies subgroups with an overall two-and-half fold increased risk of 2+ admissions (aRRR: 2.52; 95% CI: 2.25-2.82), and a 68% increased risk of a single admission (aRRR: 1.68; 1.47-1.92) compared to term infants. Preterm infants with a cardiovascular anomaly had the highest risk of repeated hospital admissions (aRRR: 3.76; 2.86-4.93) compared to term infants with a cardiovascular anomaly. Infant sex was a significant factor for 2+ admissions of infants across all anomaly subgroups. For example, girls with an anomaly had an overall 16% decreased risk of 2+ admissions, compared to boys (aRRR: 0.84; 0.78-0.91); and girls with a cardiovascular anomaly had a 25% decreased risk of 2+ admissions, compared to boys (aRRR: 0.75; 0.62-0.91). In addition, infants with the most severe and moderately severe CHD had a 2.7 (aRRR: 2.72; 1.58-4.71) and 6.3 (aRRR: 6.25; 4.47-8.74) folds increased risk of 2+ admissions

Confidential: For Review Only

respectively compared to infants with a less severe CHD. Infants with the most severe CHD had 61% reduced risk of being admitted at all (aRRR: 0.39; 0.16-0.96). For infants with multiple anomalies, those whose mothers were aged ≤24 years had a 22% increased risk of 2+ admissions (aRRR: 1.22; 1.06-1.41), while those whose mothers were aged ≥ 35 had a 46% decreased risk of 2+ admissions (aRRR: 0.54; 0.47-0.63) and a 42% decreased risk of single admissions (aRRR: 0.58; 0.48-0.69) compared to those whose mothers were aged 25-29 years. Maternal smoking was associated with an increased risk of 2+ admissions for all anomalies (aRRR: 1.20; 1.10-1.31) and all isolated anomalies (aRRR: 1.27; 1.13-1.41).

Among infants who had at least one admission, maternal age, parity, maternal smoking, and infant sex were not associated with having admissions for causes related to the anomaly as opposed to admissions for other causes (Supplementary Tables 2 and 3). However, preterm infants were less like to have anomaly-specific admissions (aOR: 0.47; 0.42-0.52), whereas infants with mothers having a history of anomalies in previous pregnancies were more likely to have anomaly-specific admissions (aOR: 1.26; 1.11-1.43).

Discussion

Using linked de-identified data from CARIS, PEDW and ONS, this is the first population-based cohort study of which we are aware to investigate a comprehensive range of risk factors associated with hospital admissions during infancy in babies born with congenital anomalies or rare diseases of congenital

Confidential: For Review Only

origin. Preterm birth was the strongest risk factor associated with an increased risk of one and two or more infant hospitalisation across all anomaly or rare disease of congenital origin subgroups. In addition, infants with two or more admissions had a higher risk profile than those with only one or no admission. Infants who had two or more admissions were more likely to be boys, born preterm, or to have mothers who were younger and smokers or for those who were born with a moderately severe CHD anomaly, compared to those with one or no admission.

Previous studies have shown that infants with a congenital anomaly were associated with a significantly high risk of hospitalisation compared to infants without an anomaly in the general population due to anomaly-related comorbidities and complications, which require ongoing inpatient treatment and investigations [6, 20-22].

Maternal and infant factors have an impact on hospitalisation in infant with congenital anomalies through different potential mechanisms. For example, previous studies have shown that teenage mothers may have an increased risk of adverse neonatal outcomes due to inadequate antenatal care and social support, and other behavioural determinants such as maternal smoking, alcohol and recreational drug misuse compared to older mothers [23]. Infants born to older mothers tend to have a reduced risk of unintentional injuries, an increased uptake of routine immunisation in childhood and may receive better parenting [24]. It has been shown that maternal smoking during pregnancy increases the risk of respiratory and infectious diseases in infants [25-27]; possible mechanisms include intrauterine stress and impaired immune system due to nicotine substances and tobacco smoking, although the exact

Confidential: For Review Only

biological mechanism is not clearly defined [28]. In our subgroup analysis, we found no significant difference in the relationship between maternal age and anomaly-specific admissions or other-cause admissions, or those between maternal smoking and anomaly-specific admissions or other-cause admissions, which suggests that the mechanisms by which maternal age and smoking are risk factors for hospitalisation of infants with an anomaly are similar for anomaly-specific admissions and admissions due to other causes.

Preterm birth has consistently been shown to be associated with an increased risk of infant morbidity and hospitalisation; preterm infants generally have a lower physiological reserve and a higher susceptibility to comorbidities and infections compared to term infants [29]. In addition, clinicians may be more cautious and adopt a lower threshold for admitting preterm infants with a congenital anomaly when complications arise [30]. Previous twin studies have shown that boys have an increased risk of respiratory distress syndrome, intrauterine growth restriction and overall morbidities compared with girls and therefore are more likely to require hospitalisation [16, 31-33]. Our subgroup analysis suggests that many infants with congenital anomalies who were born preterm required hospitalisation for conditions related to prematurity rather than the congenital anomaly, although it is possible that clinicians tend to ascribe ‘prematurity’ as the primary cause of admission for preterm babies regardless of their anomaly status.

Infants born with the most severe CHD (e.g. single ventricle and hypoplastic heart syndromes) often experience life-threatening events at birth and hence the mortality risk among these infants is considerably higher during their first admission

Confidential: For Review Only

compared to infants with less severe CHD [34, 35]. On the other hand, infants born with a moderately severe CHD are now more likely to survive due to advances in diagnostics, and medical and surgical interventions [36]; however, they are also likely to experience long-term morbidities and complications [5, 37, 38], which may explain the highest risk of repeated hospitalisation in this group of infants.

The main strengths of this study include the national study population, robust study design and data collection. Infants with congenital anomalies or rare diseases of congenital origin were identified from CARIS, an active surveillance and multiple source reporting system covering all livebirths in Wales which maximises case finding and internal validity. The use of routinely collected data from PEDW is particularly advantageous for this hospitalisation study due to the large size of the dataset and long period of data collection. A further strength relates to the broad definition of congenital anomalies, which includes structural and chromosomal defects, as well as rare diseases of congenital origin such as metabolic, endocrine and congenital blood disorders. Consequently, we were able to investigate risk factors associated with hospitalisation of infants with a wide range of congenital anomalies and rare diseases of congenital origin.

There are several limitations. As PEDW is a hospital administrative dataset, data on admissions was not always complete which can be due to infants being treated in a specialist centre outside the catchment area of NHS Wales. In addition, the number of variables explored in this study was restricted by what was available in the dataset. It is possible that institutional factors such as outpatient follow ups which

Confidential: For Review Only

were not available in this dataset are also important determinants of hospitalisation of infants with congenital anomalies.

Conclusions

Infants with congenital anomalies and rare diseases of congenital origin have a high risk of hospitalisation in infancy. Our findings can help clinicians to identify infants who are likely to require hospitalisation according to their socio-demographic and clinical characteristics. Public health interventions such as promoting good prenatal care and smoking cessation may have an important role in reducing the need for hospitalisation in infants with a congenital anomaly.

Confidential: For Review Only

What is known about the subject:

- Congenital anomalies are a leading cause of infant morbidity and hospitalisation.
- Evidence about the risk factors contributing to the hospitalisation of infants with congenital anomalies is limited.

What this study adds

- Infants who had ≥2 admissions were more likely to be boys, born preterm, born with a moderately severe CHD, or have mothers who were younger and smokers, compared to those with ≤1 admission.
- Preterm birth is the strongest risk factor for hospitalisation in infants with congenital anomalies or rare diseases of congenital origin.
- The mechanisms by which maternal age, smoking and infant sex are risk factors for hospitalisation are similar for anomaly-specific admissions and admissions due to other causes.

Confidential: For Review Only

Data sharing statement

The de-identified health data for this study was provided by the Secure Anonymised Information Linkage (SAIL) databank.

Acknowledgements

We thank the SAIL team for providing the datasets and their assistance with data linkage for this study, and the CARIS team for their support on the dataset.

Funding

This study is part-funded by the National Institute for Health Research (NIHR) Policy Research Programme, conducted through the NIHR Policy Research Unit in Maternal Health and Care, 108/0001. The views expressed are those of the author(s) and not necessarily those of the NIHR or the Department of Health and Social Care.

Peter Ho is funded by a Clarendon – Green Templeton College – Nuffield Department of Population Health Scholarship, University of Oxford.

Competing interests

None.

Confidential: For Review Only

Contributorship statement

PH contributed to the design of the study, acquisition, analysis, interpretation of data, drafting and revising it critically, approved the final version to be published, and agrees to be accountable for all aspects of the work in ensuring that questions related to the accuracy or integrity of any part of the work are appropriately investigated and resolved.

MQ and JK contributed to the design of the study, acquisition, analysis, and interpretation of data for the work; supervised the project and revised it critically; approved the final version to be published; and agree to be accountable for all aspects of the work in ensuring that questions related to the accuracy or integrity of any part of the work are appropriately investigated and resolved.

DT contributed to the acquisition, interpretation of data for the study, revised it critically, approved the final version to be published, and agrees to be accountable for all aspects of the work in ensuring that questions related to the accuracy or integrity of any part of the work are appropriately investigated and resolved.

For peer review only

Table 1: Factors associated with hospitalisation of infants with any anomaly or rare disease of congenital origin.

Characteristics	Number of admissions		
	2+<br>No. of infants (%)<br>*n = 10,785*	1<br>No. of infants (%)<br>*n = 6,043*	0<br>No. of infants (%)<br>*n = 8,695*
Socio-demographic factors			
Townsend quintile			
1 (least deprived)	1,542 (14.3)	991 (16.4)	1,272 (16.3)
2	1,645 (15.3)	956 (15.8)	1,316 (16.9)
3	1,928 (17.9)	1,102 (18.3)	1,571 (20.1)
4	2,351 (21.8)	1,257 (20.8)	1,558 (20.0)
5 (most deprived)	3,296 (30.6)	1,727 (28.6)	2,080 (26.7)
Maternal ethnicity			
White	6,729 (94.5)	3,547 (93.8)	4,252 (95.0)
Other	394 (5.5)	236 (6.2)	224 (5.0)
Maternal age at birth, years			
Mean (SD) (year)	*27.8 (6.3)*	*28.3 (6.2)*	*28.6 (6.2)*
Median (IQR)	*28 (23-32)*	*28 (24-33)*	*29 (24-33)*
≤ 24	3,596 (33.3)	1,768 (29.3)	2,204 (28.2)
25 – 29	2,880 (26.7)	1,697 (28.1)	2,096 (26.8)
30 – 34	2,585 (24.0)	1,526 (25.3)	2,046 (26.2)
≥ 35	1,720 (16.0)	1,050 (17.4)	1,468 (18.9)
Maternal factors			
Parity			
Nulliparous	4,572 (45.2)	2,454 (43.6)	3,029 (43.3)
≥ 1	5,535 (54.8)	3,173 (56.4)	3,973 (56.7)
Multiple fetus			
Yes	417 (3.9)	175 (2.9)	185 (2.4)
No	10,365 (96.1)	5,866 (97.1)	7,640 (97.6)
Maternal smoking			
Smoker	2,200 (30.7)	1,036 (27.1)	1,136 (25.5)
Non/ Ex smoker	4,958 (69.3)	2,783 (72.9)	3,315 (74.5)
Anomalies in previous pregnancies			
Yes	928 (12.0)	506 (12.2)	582 (12.0)
No	6,811 (88.0)	3,637 (87.8)	4,280 (88.0)
Infant factors			
Infant sex			
Male	6,595 (61.2)	3,599 (59.6)	5,205 (59.9)
Female	4,187 (38.8)	2,441 (40.4)	3,486 (40.1)
Birthweight, grams			
Mean (SD)	*3,024 (810)*	*3,207 (697)*	*3,286 (660)*
Median (IQR)	*3,140 (2,610-3,570)*	*3,280 (2,860-3,660)*	*3,350 (2,950-3,710)*
< 2500 (low birthweight)	2,311 (21.5)	815 (13.5)	806 (10.1)
≥ 2500	8,441 (78.5)	5,209 (86.4)	7,166 (89.9)
Gestational age, week			
Mean (SD)	*37.9 (3.3)*	38.6 (2.6)	39.0 (2.3)
Median (IQR)	*39 (37-40)*	39 (38-40)	39 (38-40)
< 37 (preterm)	2,287 (21.3)	844 (14.0)	727 (9.1)
≥ 37 (term)	8,465 (78.7)	5,178 (86.0)	7,249 (90.9)

Note: Missing data are not shown due to risk of statistical disclosure.

Confidential: For Review Only

Table 2: Univariable analyses: factors associated with repeated (2+) and single (1) admissions by anomaly/rare disease subgroup.

	2+ admissions			1 admission		
	Unadjusted RRR			Unadjusted RRR		
Factors	All	Isolated	Multiple	All	Isolated	Multiple
Townsend quintile						
1	1 (reference)	1 (reference)	1 (reference)	1 (reference)	1 (reference)	1 (reference)
2	1.03 (0.93-1.14)	1.04 (0.92-1.18)	1.06 (0.88-1.28)	0.93 (0.83-1.05)	0.88 (0.77-1.01)	1.09 (0.87-1.37)
3	1.01 (0.92-1.12)	1.06 (0.93-1.20)	0.93 (0.78-1.10)	0.90 (0.81-1.01)	0.88 (0.77-1.01)	0.92 (0.74-1.14)
4	1.24 (1.13-1.37)	1.23 (1.08-1.39)	1.24 (1.04-1.47)	1.04 (0.93-1.16)	1.02 (0.89-1.16)	1.08 (0.87-1.34)
5	1.31 (1.19-1.43)	1.32 (1.18-1.48)	1.28 (1.08-1.51)	1.07 (0.96-1.18)	1.02 (0.90-1.15)	1.17 (0.96-1.44)
Maternal ethnicity						
White	1 (reference)	1 (reference)	1 (reference)	1 (reference)	1 (reference)	1 (reference)
Other	1.11 (0.94-1.32)	1.10 (0.88-1.38)	1.06 (0.82-1.39)	1.26 (1.05-1.52)	1.34 (1.06-1.69)	1.13 (0.82-1.56)
Maternal age at delivery, years						
≤ 24	1.19 (1.10-1.28)	1.16 (1.05-1.27)	1.23 (1.07-1.41)	0.99 (0.91-1.09)	0.97 (0.87-1.07)	1.05 (0.88-1.25)
25 – 29	1 (reference)	1 (reference)	1 (reference)	1 (reference)	1 (reference)	1 (reference)
30 – 34	0.92 (0.85-1.00)	0.89 (0.81-0.98)	0.94 (0.81-1.09)	0.92 (0.84-1.01)	0.93 (0.83-1.04)	0.90 (0.75-1.07)
≥ 35	0.53 (0.49-0.58)	0.50 (0.45-0.56)	0.55 (0.48-0.64)	0.55 (0.50-0.61)	0.54 (0.48-0.61)	0.58 (0.48-0.69)
Parity						
Nulliparous	1 (reference)	1 (reference)	1 (reference)	1 (reference)	1 (reference)	1 (reference)
≥ 1	0.73 (0.69-0.77)	0.73 (0.68-0.79)	0.76 (0.68-0.84)	0.78 (0.73-0.84)	0.78 (0.72-0.85)	0.80 (0.71-0.91)
Multiple fetus						
Yes	1.66 (1.39-1.98)	1.92 (1.53-2.39)	1.20 (0.89-1.60)	1.23 (1.00-1.52)	1.43 (1.11-1.84)	0.87 (0.60-1.27)
No	1 (reference)	1 (reference)	1 (reference)	1 (reference)	1 (reference)	1 (reference)
Maternal smoking						
Smoker	1.30 (1.19-1.41)	1.32 (1.19-1.48)	1.24 (1.09-1.43)	1.09 (0.99-1.20)	1.06 (0.94-1.20)	1.14 (0.96-1.34)
Non/Ex smoker	1 (reference)	1 (reference)	1 (reference)	1 (reference)	1 (reference)	1 (reference)
Anomalies in previous pregnancies						
Yes	1.00 (0.89-1.11)	1.04 (0.91-1.20)	0.81 (0.67-0.99)	0.98 (0.86-1.11)	1.00 (0.86-1.16)	0.90 (0.70-1.14)
No	1 (reference)	1 (reference)	1 (reference)	1 (reference)	1 (reference)	1 (reference)
Infant sex[1]						
Male	1 (reference)	1 (reference)	1 (reference)	1 (reference)	1 (reference)	1 (reference)
Female	0.95 (0.90-1.00)	0.94 (0.87-1.01)	0.88 (0.79-0.97)	1.01 (0.95-1.08)	1.07 (0.99-1.16)	0.86 (0.76-0.97)
Gestational age[1], week						
< 37	2.95 (2.70-3.23)	3.25 (2.89-3.64)	2.15 (1.87-2.49)	1.79 (1.61-1.98)	1.91 (1.67-2.18)	1.48 (1.24-1.77)
≥ 37	1 (reference)	1 (reference)	1 (reference)	1 (reference)	1 (reference)	1 (reference)

[1]Variable identified for inclusion a priori. Note: Statistical significance at p < 0.1.

Confidential: For Review Only

Table 3: Multivariable analyses: factors associated with repeated (2+) and single (1) admissions by anomaly or rare disease of congenital origin subgroup.

	2+ admissions			1 admission		
	Adjusted RRR[2]			Adjusted RRR[2]		
Factors	All	Isolated	Multiple	All	Isolated	Multiple
Maternal age at delivery, years						
≤ 24	1.17 (1.06-1.30)		1.22 (1.06-1.41)	1.00 (0.89-1.12)		1.04 (0.88-1.24)
25 – 29	1 (reference)		1 (reference)	1 (reference)		1 (reference)
30 – 34	0.99 (0.89-1.10)		0.94 (0.81-1.09)	0.94 (0.83-1.06)		0.90 (0.75-1.07)
≥ 35	0.93 (0.82-1.04)		0.54 (0.47-0.63)	0.95 (0.83-1.09)		0.58 (0.48-0.69)
Maternal smoking						
Smoker	1.20 (1.10-1.31)	1.26 (1.13-1.41)		1.06 (0.96-1.17)	1.04 (0.92-1.18)	
Non/Ex smoker	1 (reference)	1 (reference)		1 (reference)	1 (reference)	
Infant sex[1]						
Male	1 (reference)	1 (reference)	1 (reference)	1 (reference)	1 (reference)	1 (reference)
Female	0.84 (0.78-0.91)	0.82 (0.74-0.90)	0.90 (0.81-1.00)	0.95 (0.87-1.04)	1.01 (0.91-1.12)	0.87 (0.77-0.99)
Gestational age[1], week						
< 37	2.52 (2.25-2.83)	2.83 (2.41-3.31)	2.19 (1.90-2.53)	1.68 (1.47-1.92)	1.85 (1.55-2.21)	1.50 (1.26-1.79)
≥ 37	1 (reference)	1 (reference)	1 (reference)	1 (reference)	1 (reference)	1 (reference)

[1]*Variable identified for inclusion a priori.*
[2]*For 'All' anomaly subgroup, adjusted variables include maternal age at delivery, maternal smoking, infant sex and gestational age; for 'isolate' anomaly subgroup, adjusted variables include maternal smoking, infant sex and gestation age; for 'multiple' anomaly subgroup, adjusted variables include maternal age at delivery, infant sex and gestational age. Statistical significance at $p < 0.05$.*

Confidential: For Review Only

Table 4: Unadjusted and adjusted factors associated with repeated (2+) and single (1) admissions of infants with cardiovascular anomalies.

	2+ admissions		1 admission	
	Cardiovascular			
Factors	Unadjusted[2]	Adjusted[3]	Unadjusted[2]	Adjusted[3]
Townsend quintile				
1	1 (reference)		1 (reference)	
2	1.11 (0.80-1.53)		0.80 (0.56-1.12)	
3	1.35 (0.99-1.85)		0.94 (0.68-1.31)	
4	1.10 (0.81-1.50)		1.05 (0.77-1.43)	
5	1.40 (1.05-1.85)		0.97 (0.72-1.30)	
Maternal ethnicity				
White	1 (reference)		1 (reference)	
Other	0.82 (0.49-1.38)		0.91 (0.53-1.57)	
Maternal age at delivery, years				
≤ 24	1.20 (0.95-1.53)		0.94 (0.72-1.23)	
25 – 29	1 (reference)		1 (reference)	
30 – 34	1.02 (0.80-1.31)		1.08 (0.83-1.41)	
≥ 35	0.57 (0.43-0.75)		0.67 (0.51-0.90)	
Parity				
Nulliparous	1 (reference)		1 (reference)	
≥ 1	0.92 (0.76-1.11)		0.86 (0.70-1.05)	
Multiple fetus				
Yes	1.86 (1.15-3.00)		1.55 (0.91-2.62)	
No	1 (reference)		1 (reference)	
Maternal smoking				
Smoker	1.39 (1.07-1.79)		1.07 (0.81-1.42)	
Non/Ex smoker	1 (reference)		1 (reference)	
Anomalies in previous pregnancies				
Yes	1.32 (0.94-1.86)		1.20 (0.84-1.71)	
No	1 (reference)		1 (reference)	
Infant sex[1]				
Male	1 (reference)	1 (reference)	1 (reference)	1 (reference)
Female	0.66 (0.55-0.79)	0.75 (0.62-0.91)	0.95 (0.78-1.15)	0.95 (0.78-1.15)
Gestational age[1], week				
< 37	3.33 (2.55-4.35)	3.76 (2.86-4.93)	1.97 (1.46-2.65)	1.97 (1.46-2.66)
≥ 37	1 (reference)	1 (reference)	1 (reference)	1 (reference)
CHD Disease Severity[1]				
Less severity	1 (reference)	1 (reference)	1 (reference)	1 (reference)
Moderate severity	5.83 (4.19-8.11)	6.25 (4.47-8.74)	1.14 (0.75-1.75)	1.18 (0.77-1.81)
Most severe	2.67 (1.57-4.57)	2.72 (1.58-4.71)	0.38 (0.15-0.94)	0.39 (0.16-0.96)

[1]Variable identified for inclusion a priori. [2]Statistically significant at $p < 0.1$. [3]Adjusted variables include infant sex, gestational age and disease severity. Statistically significant at $p < 0.05$.

Confidential: For Review Only

References

1. Yoon, P.W., Olney, R.S., Khoury, M.J., Sappenfield, W.M., Chavez, G.F. and Taylor, D., 1997. Contribution of birth defects and genetic diseases to pediatric hospitalizations: a population-based study. *Archives of pediatrics & adolescent medicine*, *151*(11), pp.1096-1103.
2. Cherian, J., Staggers, K.A., Pan, I.W., Lopresti, M., Jea, A. and Lam, S., 2016. Thirty-day outcomes after postnatal myelomeningocele repair: a National Surgical Quality Improvement Program Pediatric database analysis. *Journal of Neurosurgery: Pediatrics*, *18*(4), pp.416-422.
3. Bradnock, T.J., Marven, S., Owen, A., Johnson, P., Kurinczuk, J.J., Spark, P., Draper, E.S. and Knight, M., 2011. Gastroschisis: one year outcomes from national cohort study. *Bmj*, *343*, p.d6749.
4. Derrington, T.M., Kotelchuck, M., Plummer, K., Cabral, H., Lin, A.E., Belanoff, C., Shin, M., Correa, A. and Grosse, S.D., 2013. Racial/ethnic differences in hospital use and cost among a statewide population of children with Down syndrome. *Research in developmental disabilities*, *34*(10), pp.3276-3287.
5. Marston, A.P., Patel, T., Nguyen, S.A. and White, D.R., 2019. Short-Term Risk Factor Profile of Pediatric Choanal Atresia Repair Using ACS-NSQIP National Database. *Annals of Otology, Rhinology & Laryngology*, *128*(9), pp.855-861.
6. Medrano, C., Garcia-Guereta, L., Grueso, J., Insa, B., Ballesteros, F., Casaldaliga, J., Cuenca, V., Escudero, F., de la Calzada, L.G., Luis, M. and Luque, M., 2007. Respiratory infection in congenital cardiac disease. Hospitalizations in young children in Spain during 2004 and 2005: the CIVIC Epidemiologic Study. *Cardiology in the Young*, *17*(4), pp.360-371.
7. Fitzsimons, K.J., Copley, L.P., Deacon, S.A. and van der Meulen, J.H., 2013. Hospital care of children with a cleft in England. *Archives of disease in childhood*, *98*(12), pp.970-974.
8. Razzaghi, H., Dawson, A., Grosse, S.D., Allori, A.C., Kirby, R.S., Olney, R.S., Correia, J. and Cassell, C.H., 2015. Factors associated with high hospital resource use in a population-based study of children with orofacial clefts. *Birth Defects Research Part A: Clinical and Molecular Teratology*, *103*(2), pp.127-143.

Confidential: For Review Only

9. Congenital Anomaly Register and Information Service (CARIS) 2018, *Introduction. Congenital Anomaly Register and information Service*, CARIS, viewed 8 March 2019, <http://www.caris.wales.nhs.uk/introduction>.

10. National Health Service (NHS) Wales Information Service 2020, *PEDW Data Online*, NHS Wales Information Service, viewed 28 December 2019, <http://www.infoandstats.wales.nhs.uk/page.cfm?orgid=869&pid=40977>.

11. Office for National Statistics (ONS) 2015, *Statistical bulletin: Birth Summary Tables, England and Wales: 2014,* ONS, viewed 8 September 2019, <https://www.ons.gov.uk/peoplepopulationandcommunity/birthsdeathsandmarriages/livebirths/bulletins/birthsummarytablesenglandandwales/2015-07-15>.

12. NHS Wales Digital Health and Care Wales, 2021, *NHS Wales Data Dictionary,* NHS Wales Digital Health and Care Wales, viewed 23 May 2021, <http://www.datadictionary.wales.nhs.uk/>.

13. European Surveillance of Congenital Anomalies (EUROCAT) 2013, EUROCAT Guide 1.4: Instruction for the registration of congenital anomalies. EUROCAT Central Registry, University of Ulster. Last update version 28/12/2018, viewed 25 August 2019, <https://eu-rd-platform.jrc.ec.europa.eu/sites/default/files/Full_Guide_1_4_version_28_DEC2018.pdf>.

14. World Health Organization (WHO), 2004. ICD-10: international statistical classification of diseases and related health problems: tenth revision.

15. Butler, A.S. and Behrman, R.E. eds., 2007. *Preterm birth: causes, consequences, and prevention*. National academies press.

16. Stevenson, D.K., Verter, J., Fanaroff, A.A., Oh, W., Ehrenkranz, R.A., Shankaran, S., Donovan, E.F., Wright, L.L., Lemons, J.A., Tyson, J.E. and Korones, S.B., 2000. Sex differences in outcomes of very low birthweight infants: the newborn male disadvantage. *Archives of disease in childhood-fetal and neonatal edition*, *83*(3), pp.F182-F185.

17. Islam, S., Yasui, Y., Kaul, P., Marelli, A.J. and Mackie, A.S., 2016. Congenital heart disease hospitalizations in Canada: a 10-year experience. *Canadian Journal of Cardiology*, *32*(2), pp.197-203.

Confidential: For Review Only

18. Mackie, A.S., Tran, D.T., Marelli, A.J. and Kaul, P., 2017. Cost of congenital heart disease hospitalizations in Canada: a population-based study. *Canadian Journal of Cardiology*, *33*(6), pp.792-798.
19. StataCorp. 2013. *Stata Statistical Software: Release 13*. College Station, TX: StataCorp LP.
20. Colvin, L. and Bower, C., 2009. A retrospective population-based study of childhood hospital admissions with record linkage to a birth defects registry. *BMC pediatrics*, *9*(1), p.32.
21. Wynn, J., Krishnan, U., Aspelund, G., Zhang, Y., Duong, J., Stolar, C.J., Hahn, E., Pietsch, J., Chung, D., Moore, D. and Austin, E., 2013. Outcomes of congenital diaphragmatic hernia in the modern era of management. *The Journal of pediatrics*, *163*(1), pp.114-119.
22. Frid, C., Annerén, G., Rasmussen, F., Sundelin, C. and Drott, P., 2002. Utilization of medical care among children with Down's syndrome. *Journal of Intellectual Disability Research*, *46*(4), pp.310-317.
23. Harper, L.M., Chang, J.J. and Macones, G.A., 2011. Adolescent pregnancy and gestational weight gain: do the Institute of Medicine recommendations apply?. *American journal of obstetrics and gynecology*, *205*(2), pp.140-e1.
24. Sutcliffe, A.G., Barnes, J., Belsky, J., Gardiner, J. and Melhuish, E., 2012. The health and development of children born to older mothers in the United Kingdom: observational study using longitudinal cohort data. *Bmj*, *345*, p.e5116.
25. DiFranza, J.R., Aligne, C.A. and Weitzman, M., 2004. Prenatal and postnatal environmental tobacco smoke exposure and children's health. *Pediatrics*, *113*(Supplement 3), pp.1007-1015.
26. Håberg, S.E., Stigum, H., Nystad, W. and Nafstad, P., 2007. Effects of pre-and postnatal exposure to parental smoking on early childhood respiratory health. *American journal of epidemiology*, *166*(6), pp.679-686.
27. Yusuf, H.R., Rochat, R.W., Baughman, W.S., Gargiullo, P.M., Perkins, B.A., Brantley, M.D. and Stephens, D.S., 1999. Maternal cigarette smoking and invasive meningococcal disease: a cohort study among young children in metropolitan Atlanta, 1989-1996. *American journal of public health*, *89*(5), pp.712-717.

Confidential: For Review Only

28. Weinsheimer, R.L. and Yanchar, N.L., 2008. Impact of maternal substance abuse and smoking on children with gastroschisis. *Journal of pediatric surgery*, *43*(5), pp.879-883.

29. Björ, O. and Bråbäck, L., 2003. A retrospective population based trend analysis on hospital admissions for lower respiratory illness among Swedish children from 1987 to 2000. *BMC Public Health*, *3*(1), p.22.

30. Mackie, A.S., Ionescu-Ittu, R., Pilote, L., Rahme, E. and Marelli, A.J., 2008. Hospital readmissions in children with congenital heart disease: a population-based study. *American heart journal*, *155*(3), pp.577-584.

31. Lisi, A., Botto, L.D., Rittler, M., Castilla, E., Bianchi, F., Botting, B., De Walle, H., Erickson, J.D., Gatt, M., De Vigan, C. and Irgens, L., 2005. Sex and congenital malformations: an international perspective. *American Journal of Medical Genetics Part A*, *134*(1), pp.49-57.

32. Mulla, Z.D., Plavsic, S.K., Ortiz, M., Nuwayhid, B.S. and Ananth, C.V., 2013. Fetal sex pairing and adverse perinatal outcomes in twin gestations. *Annals of epidemiology*, *23*(1), pp.7-12.

33. Kent, A.L., Wright, I.M. and Abdel-Latif, M.E., 2012. Mortality and adverse neurologic outcomes are greater in preterm male infants. *Pediatrics*, *129*(1), pp.124-131.

34. van Dongen, E.I., Glansdorp, A.G., Mildner, R.J., McCrindle, B.W., Sakopoulos, A.G., VanArsdell, G., Williams, W.G. and Bohn, D., 2003. The influence of perioperative factors on outcomes in children aged less than 18 months after repair of tetralogy of Fallot. *The Journal of thoracic and cardiovascular surgery*, *126*(3), pp.703-710.

35. Desai, J., Aggarwal, S., Lipshultz, S., Agarwal, P., Yigazu, P., Patel, R., Seals, S. and Natarajan, G., 2017. Surgical Interventions in Infants Born Preterm with Congenital Heart Defects: An Analysis of the Kids' Inpatient Database. *The Journal of pediatrics*, *191*, pp.103-109.

36. Holst, K.A., Said, S.M., Nelson, T.J., Cannon, B.C. and Dearani, J.A., 2017. Current interventional and surgical management of congenital heart disease: specific focus on valvular disease and cardiac arrhythmias. *Circulation research*, *120*(6), pp.1027-1044.

37. Smith, A.H., Doyle, T.P., Mettler, B.A., Bichell, D.P. and Gay, J.C., 2015. Identifying predictors of hospital readmission following congenital heart surgery through

Confidential: For Review Only

analysis of a multiinstitutional administrative database. *Congenital heart disease*, *10*(2), pp.142-152.

38. Duppenthaler, A., Ammann, R.A., Gorgievski-Hrisoho, M., Pfammatter, J.P. and Aebi, C., 2004. Low incidence of respiratory syncytial virus hospitalisations in haemodynamically significant congenital heart disease. *Archives of disease in childhood*, *89*(10), pp.961-965.

Confidential: For Review Only

Supplementary Table 1: Inclusion of anomaly and rare disease of congenital origin subgroups.

Subgroup	Classification of anomaly and rare disease of congenital origin included
All anomalies; all isolated anomalies; and all multiple anomalies	• Nervous system;<br>• Eye, ear, face and neck;<br>• Cardiovascular system;<br>• Respiratory and clefts;<br>• Abdominal wall defects and diaphragmatic hernia;<br>• Upper and lower gastrointestinal;<br>• Genitourinary;<br>• Limb and skeletal;<br>• Blood disorders;<br>• Endocrine and Metabolic;<br>• Neoplasm;<br>• Syndromes and congenital malformation syndromes;<br>• Maternal Infection leading to malformations.
Cardiovascular anomalies	• Congenital heart defects and diseases of the circulatory system.

Confidential: For Review Only

Supplementary Table 2: Factors associated with cause-specific admissions among infants with any anomaly or rare disease of congenital origin.

	Anomaly-specific admissions, number of infants (%)	Other-cause admissions, number of infants (%)	Unadjusted OR (95% CI)	P-value[1]
Socio-demographic factors				
Townsend quintile				
1 (least deprived)	772 (15.4)	1,709 (14.9)	1 (reference)	0.253
2	767 (15.3)	1,780 (15.6)	0.95 (0.85-1.08)	
3	932 (18.6)	2,042 (17.9)	1.01 (0.90-1.13)	
4	1,093 (21.8)	2,432 (21.3)	0.99 (0.89-1.11)	
5 (most deprived)	1,439 (28.8)	3,472 (30.4)	0.92 (0.83-1.02)	
Maternal ethnicity				
White	3,101 (94.2)	6,948 (94.2)	1 (reference)	0.978
Other	191 (5.8)	429 (5.8)	1.00 (0.84-1.19)	
Maternal age at birth, years *(n = 29,400)*				
≤ 24	1,570 (31.3)	3,680 (32.1)	0.97 (0.89-1.06)	0.712
25 – 29	1,363 (27.1)	3,103 (27.1)	1 (reference)	
30 – 34	1,251 (24.9)	2,782 (24.3)	1.02 (0.93-1.22)	
≥ 35	831 (16.6)	1,891 (16.5)	1.00 (0.90-1.11)	
Maternal factors				
Parity				
Nulliparous	2,101 (41.9)	4,782 (41.7)	1 (reference)	0.856
≥ 1	2,914 (58.1)	6,674 (58.3)	0.99 (0.93-1.06)	
Multiple fetus				
Yes	153 (3.1)	427 (3.7)	0.81 (0.67-0.98)	0.028
No	4,862 (96.9)	11,029 (96.3)	1 (reference)	
Maternal smoking				
Smoker	946 (28.0)	2,218 (30.1)	0.90 (0.83-0.99)	0.027
Non/ Ex smoker	2,430 (72.0)	5,147 (69.9)	1 (reference)	
Anomalies in previous pregnancies				
Yes	374 (10.4)	1,028 (12.8)	1.27 (1.12-1.44)	<0.001
No	3,231 (89.6)	6,981 (87.2)	1 (reference)	
Infant factors				
Infant sex				
Male	3,013 (60.1)	6,964 (60.8)	1 (reference)	0.405
Female	1,999 (39.9)	4,489 (39.2)	1.03 (0.96-1.10)	
Birthweight, grams				
< 2500	598 (11.9)	2,448 (21.4)	0.50 (0.45-0.55)	<0.0001
≥ 2500	4,417 (88.0)	9,008 (78.6)	1 (reference)	
Gestational age, week				
$< 37^{+0}$	577 (11.5)	2,470 (21.6)	0.47 (0.43-0.52)	<0.0001
$\geq 37^{+0}$	4,438 (88.5)	8,986 (78.4)	1 (reference)	

[1]P < 0.1 was considered as statistically significant. Missing data are not shown due to risk of statistical disclosure.

Confidential: For Review Only

Supplementary Table 3: Factors associated with cause-specific admissions among infants with any anomaly or rare disease of congenital origin: multivariable analysis.

Risk factors	Crude OR (95% CI)	P-value[1]	aOR[2] (95% CI)	P-value[3]
Anomalies in previous pregnancies				
Yes	1.27 (1.12-1.44)	<0.001	1.26 (1.11-1.43)	<0.001
No	1 (reference)		1 (reference)	
Gestational age, week				
< 37 (preterm)	0.47 (0.43-0.52)	<0.0001	0.47 (0.42-0.52)	<0.0001
≥ 37 (term)	1 (reference)		1 (reference)	

[1]P-value of the univariable analysis < 0.1 was considered as statistically significant. [2]Mutually adjusted odds ratios for other factors in the table. [3]P-value of the multivariable analysis < 0.05 was considered as statistically significant.

Confidential: For Review Only

BMJ Paediatrics Open

Risk factors for hospitalisation in Welsh infants with a congenital anomaly

Journal:	*BMJ Paediatrics Open*
Manuscript ID	bmjpo-2021-001238.R1
Article Type:	Original research
Date Submitted by the Author:	26-Oct-2021
Complete List of Authors:	Ho, Peter ; University of Oxford, Nuffield Department of Population Health<br>Quigley, Maria; University of Oxford, National Perinatal Epidemiology Unit, Nuffield Department of Population Health; University of Oxford, National Institute for Health Research (NIHR) Policy Research Unit-Maternal Health and Care, National Perinatal Epidemiology Unit, Nuffield Department of Population Health<br>Tucker, David; Public Health Wales, Congenital Anomaly Register and Information Service (CARIS) for Wales<br>Kurinczuk, Jenny; University of Oxford, National Perinatal Epidemiology Unit, Nuffield Department of Population Health; University of Oxford, National Institute for Health Research (NIHR) Policy Research Unit-Maternal Health and Care, National Perinatal Epidemiology Unit, Nuffield Department of Population Health
Keywords:	Epidemiology, Neonatology, Health services research

SCHOLARONE™
Manuscripts

I, the Submitting Author has the right to grant and does grant on behalf of all authors of the Work (as defined in the below author licence), an exclusive licence and/or a non-exclusive licence for contributions from authors who are: i) UK Crown employees; ii) where BMJ has agreed a CC-BY licence shall apply, and/or iii) in accordance with the terms applicable for US Federal Government officers or employees acting as part of their official duties; on a worldwide, perpetual, irrevocable, royalty-free basis to BMJ Publishing Group Ltd ("BMJ") its licensees and where the relevant Journal is co-owned by BMJ to the co-owners of the Journal, to publish the Work in this journal and any other BMJ products and to exploit all rights, as set out in our licence.

The Submitting Author accepts and understands that any supply made under these terms is made by BMJ to the Submitting Author unless you are acting as an employee on behalf of your employer or a postgraduate student of an affiliated institution which is paying any applicable article publishing charge ("APC") for Open Access articles. Where the Submitting Author wishes to make the Work available on an Open Access basis (and intends to pay the relevant APC), the terms of reuse of such Open Access shall be governed by a Creative Commons licence – details of these licences and which Creative Commons licence will apply to this Work are set out in our licence referred to above.

Other than as permitted in any relevant BMJ Author's Self Archiving Policies, I confirm this Work has not been accepted for publication elsewhere, is not being considered for publication elsewhere and does not duplicate material already published. I confirm all authors consent to publication of this Work and authorise the granting of this licence.

Confidential: For Review Only

RISK FACTORS FOR HOSPITALISATION IN WELSH INFANTS WITH A CONGENITAL ANOMALY.

Peter Ho[1], Maria A Quigley[1,2], David Tucker[3], Jennifer J Kurinczuk[1,2]

[1]National Perinatal Epidemiology Unit, Nuffield Department of Population Health, University of Oxford.

[2]National Institute for Health Research (NIHR) Policy Research Unit- Maternal Health and Care, National Perinatal Epidemiology Unit, Nuffield Department of Population Health, University of Oxford.

[3]Congenital Anomaly Register and Information Service (CARIS) in Wales.

Correspondence author: Dr. Peter Ho, National Perinatal Epidemiology Unit, Nuffield Department of Population Health, University of Oxford, Old Road Campus, Headington, Oxford OX3 7LF, UK. Email: Peterhosy@hotmail.com Tel: +44 7905497825 Fax: +44 1865 289701

Word count: 2500 (excluding abstract and references)

Confidential: For Review Only

Abstract

Objectives: To investigate risk factor associated with hospitalisation of infants with a congenital anomaly in Wales, UK.

Design: A population-based cohort study.

Setting: Data from the Welsh Congenital Anomaly Register and Information Service (CARIS) linked to the Patient Episode Database for Wales (PEDW) and livebirths and deaths from the Office for National Statistics (ONS).

Patients: All livebirths between 1999 and 2015 with a diagnosis of a congenital anomaly, which was defined as a structural, metabolic, endocrine, or genetic defect, as well as rare diseases of hereditary origin.

Main outcome measures: Adjusted relative risk ratios (aRRR) associated with 1 or 2+ hospital admissions in infancy versus no admissions were estimated for socio-demographic, maternal and infant factors using multinomial logistic regression for the subgroups of all, isolated, multiple and cardiovascular anomalies.

Results: 25,523 infants affected by congenital anomalies experienced a total of 50,705 admissions in infancy. Risk factors for ≥2 admissions were younger maternal age ≤24 years (aRRR: 1.17; 95% CI: 1.06-1.30), maternal smoking (aRRR: 1.20; 1.10-1.31), preterm birth (aRRR: 2.52; 2.25-2.82) and moderately severe congenital heart defects (aRRR: 6.25; 4.47-8.74). Girls had an overall decreased risk of 2+ admissions (aRRR: 0.84; 0.78-0.91). Preterm birth was a significant risk factor for admissions in

Confidential: For Review Only

all anomaly subgroups but the effect of the other characteristics varied according to anomaly subgroup.

Conclusions: Over two thirds of infants with an anomaly are admitted to hospital during infancy. Our findings identified socio-demographic and clinical characteristics contributing to an increased risk of hospitalisation of infants with congenital anomalies.

Confidential: For Review Only

Introduction

Congenital anomalies are a leading cause of infant morbidity and hospitalisation [1]. Infants who are born with a major congenital anomaly such as a congenital heart defect (CHD), Down syndrome and congenital diaphragmatic hernia often experience anomaly-related sequelae and complications, and require more frequent hospitalisation compared to children without an anomaly.

There is some evidence that infant characteristics can influence hospitalisation of children affected by specific anomalies. For example, previous studies have shown that preterm birth, low birthweight, disease severity and co-morbidity are significantly associated with an increased risk of excess hospitalisation of infants with spina bifida, choanal atresia, congenital heart defects, orofacial clefts, congenital diaphragmatic hernia, biliary atresia, gastroschisis and Down syndrome [2-8]; while the effects of socio-demographic and maternal are less well established. Further research on risk factors for hospitalisation, particularly those associated with a wider range of congenital anomalies are needed to inform planning of healthcare and social interventions aimed at reducing infant morbidity and hospitalisation.

We aimed to investigate risk factors for hospitalisation in the first year after birth among infants with congenital anomalies.

Confidential: For Review Only

Methods

Study design and record linkage

A national population-based cohort study was established using registry data from the Congenital Anomaly Register and Information Service (CARIS) for Wales, linked to Patient Episode Database for Wales (PEDW), and births and deaths registration data from the Office for National Statistics (ONS) [9-11], and de-identified for analysis.

Deterministic and probabilistic linkage between datasets were performed using an unique Anonymised Linkage Field (ALF) encrypted code provided by NHS Wales Informatics Service, based on infant's NHS number, name, date of birth and address. All infants in the CARIS and PEDW datasets have a validated ALF and hence each infant was matched with hospital admissions using one to multiple merge; unmatched infants were treated as zero admissions. The outputs of linkage were validated by the Secure Anonymised Information Linkage (SAIL) team.

Study population

The inclusion criteria for this study were:

1. All livebirths between 1999 and 2015 with birthweight ≥500g, gestational age $\geq 22^{+0}$ weeks, with a confirmed or probable diagnosis of a congenital anomaly reported to CARIS. CARIS defines congenital anomalies as structural, metabolic, endocrine, or genetic defects, as well as rare diseases of hereditary origin present in the child or fetus at the end of pregnancy, even if not detected until

Confidential: For Review Only

after birth [9]. Therefore, the 10th revision of International Classification of Diseases and Related Health Problems (ICD-10) ‘Q’, ‘P35’ and ‘P37” codes, as well as other non ‘Q’ ICD-10 codes of congenital anomalies and rare diseases were also included in this study.

2. All hospital admissions (i.e. emergency, elective and transfer admissions) of infants with a congenital anomaly. An admission is defined as a hospital stay of a patient using a bed on premises controlled by the one health care provider [12]. An admission is ended by a discharge, transfer to another NHS provider or death. All infants who were admitted from birth due to medical reasons were included in the analysis.

Exclusion criteria

1. Admissions solely for the birth of babies (i.e. birth hospitalisation) were excluded.

Categorisation of anomaly

Congenital anomalies were categorised according to the European Surveillance of Congenital Anomalies (EUROCAT) subgroup classification [13]; other rare diseases which are not included in the EUROCAT subgroup classification were categorised according to the ICD chapter headings [14] (Supplementary Table 1).

Infants were considered as having an isolated congenital anomaly (or disease) if a single anomaly (or disease) was diagnosed and reported to CARIS. Infants were considered as having multiple anomalies (or diseases) if more than one anomaly (or

Confidential: For Review Only

diseases) was diagnosed, either within the same body system or involving different body systems. Infants who were diagnosed with a syndrome involving more than one anomaly (or disease) were considered as having multiple anomalies (or diseases).

Outcome

The outcome was total hospital admissions for any cause in infancy (period from birth up to the first birthday) and infants were categorised as having 0, 1 or 2+ admissions.

Statistical analysis

Multinomial logistic regression was conducted to estimate the adjusted relative risk ratios (aRRR) of hospital admission and the corresponding 95% confidence interval (CI) according to the categories of socio-demographic, maternal and infant factors (Table 1), comparing infants who had one or 2+ admissions versus those who had no admission (the comparison group). Any hospital transfers were treated as separate admissions in order to assess the full burden and utilisation of hospitalisation.

The modeling strategy aimed to identify the strongest risk factors for hospital admission. Variables included *a priori* in the multivariable models were infant sex, gestational age at birth and disease severity for cardiovascular anomalies as these are known determinants of infant morbidity and hospitalisation [5, 15-18], and these variables were included in the final model regardless of their statistical significance in the univariable analysis. The remaining variables that were associated with

Confidential: For Review Only

hospitalisation in univariable analysis ($p < 0.1$) were also initially included in multivariable models to observe their effect size and statistical significance and they were removed from the multivariable model if no category of the variable improved the fit of the data (i.e. $p < 0.05$). As birthweight and gestational age at birth were highly collinear (correlation coefficient $r = 0.75$), only gestational age at birth was included in the final model due to its greater clinical utility. All multivariable analysis was carried out separately for the following subgroups: infants with any anomaly; infants with isolated anomalies; infants with multiple anomalies; and infants with cardiovascular anomalies.

A further subgroup analysis was conducted for those babies who had at least one admission to investigate whether risk factors were different between infants with anomaly-specific admissions and those whose admissions were for 'other-causes'. Here, anomaly-specific admissions were defined as hospitalisation of infants due to 'congenital anomalies, deformations, and chromosomal abnormalities' (ICD-10 codes: Q00-Q99/ chapter XVII) as the primary reason for admission. Crude odds ratios with corresponding 95% CIs were estimated for each explanatory variable comparing these two groups of infants. Significant variables from the univariable analysis ($p < 0.1$) were included in the multivariable analysis. $P < 0.05$ was considered as statistically significant in all final models.

A sensitivity analysis was conducted by adding year of birth to the final model for each subgroup to investigate if the effect of the key risk factors changed.

Confidential: For Review Only

Missing data for the majority of variables were few and they are not shown due to the risk of statistical disclosure. All analyses were performed using complete case analysis. All statistical analyses were performed using Stata 13 [19].

Research ethics and statistical disclosure

The secondary anonymised health data for this study was provided by the SAIL databank in Wales. This study was approved by the SAIL Information Governance Review Panel (IGRP) and did not require separate research ethics committee review. All statistical disclosure control and checks were strictly followed according to the SAIL Databank and ONS guidelines, to ensure that individual infants were not deductively identifiable.

Patient and public involvement

All proposals to use data within the SAIL Databank are subject to review by an independent Information Governance Review Panel (IGRP) for privacy risk, data governance and public benefit assessment. The IGRP is made up of a range of independent experts as well as members of the public.

Results

Between 1999 and 2016, 25,523 babies were born alive and affected by congenital anomalies or rare disease of congenital origin, and required a total of 50,705 admissions in infancy. Overall, approximately a quarter of infants had only one admission, over 40% had two or more admissions and more than one third had no

Confidential: For Review Only

admission in infancy. Among infants with isolated anomalies, the largest number of admissions occurred in infants with a cardiovascular anomaly (8% of all admissions of babies with an anomaly).

Table 1 shows that infants who had two or more admissions compared with those who had one or no admission were more likely to live in a deprived area (30.6% versus 28.6% or 26.7%), to have mothers who were younger (33.3% versus 29.3% or 28.2%), nulliparous (45.2% versus 43.6% or 43.3%), and smokers (30.7% versus 27.1% or 25.5%), to be boys (61.2% versus 59.6% or 59.9%), born preterm (21.3% versus 14.0% or 9.1%), to have a low birthweight (21.5% versus 13.5% or 10.1%), or to be one of a multiple pregnancy (3.9% versus 2.9% or 2.4%).

In the univariable analysis, social deprivation, maternal age, parity, maternal smoking, and preterm birth were significantly associated with an increased or decreased risk for repeated admissions for 'all', 'all isolated' and 'all multiple' anomaly subgroups (Table 2). Except for social deprivation and maternal smoking, the same variables (i.e. maternal age and preterm births) which were associated with an increased risk for repeated admissions were also consistently associated with an increased or decreased risk of single admissions of infants for these anomaly subgroups.

Tables 3 and 4 shows the adjusted RRRs for variables significantly associated with ($p < 0.05$) the number of admissions in infancy by different anomaly or rare disease of congenital origin subgroups. Preterm birth was consistently the strongest risk factor associated with increased 1 and 2+ admissions of infants across all anomalies

Confidential: For Review Only

subgroups with an overall two-and-half fold increased risk of 2+ admissions (aRRR: 2.52; 95% CI: 2.25-2.82), and a 68% increased risk of a single admission (aRRR: 1.68; 1.47-1.92) compared to term infants. Preterm infants with a cardiovascular anomaly had the highest risk of repeated hospital admissions (aRRR: 3.76; 2.86-4.93) compared to term infants with a cardiovascular anomaly. Infant sex was a significant factor for 2+ admissions of infants across all anomaly subgroups. For example, girls with an anomaly had an overall 16% decreased risk of 2+ admissions, compared to boys (aRRR: 0.84; 0.78-0.91); and girls with a cardiovascular anomaly had a 25% decreased risk of 2+ admissions, compared to boys (aRRR: 0.75; 0.62-0.91). In addition, infants with the most severe and moderately severe CHD had a 2.7 (aRRR: 2.72; 1.58-4.71) and 6.3 (aRRR: 6.25; 4.47-8.74) folds increased risk of 2+ admissions respectively compared to infants with a less severe CHD. Infants with the most severe CHD had 61% reduced risk of being admitted at all (aRRR: 0.39; 0.16-0.96). For infants with multiple anomalies, those whose mothers were aged ≤24 years had a 22% increased risk of 2+ admissions (aRRR: 1.22; 1.06-1.41), while those whose mothers were aged ≥ 35 had a 46% decreased risk of 2+ admissions (aRRR: 0.54; 0.47-0.63) and a 42% decreased risk of single admissions (aRRR: 0.58; 0.48-0.69) compared to those whose mothers were aged 25-29 years. Maternal smoking was associated with an increased risk of 2+ admissions for all anomalies (aRRR: 1.20; 1.10-1.31) and all isolated anomalies (aRRR: 1.27; 1.13-1.41). The effects of each variable did not vary substantially when adjusted for year of birth (Supplementary Table 2).

Confidential: For Review Only

Among infants who had at least one admission, maternal age, parity, maternal smoking, and infant sex were not associated with having admissions for causes related to the anomaly as opposed to admissions for other causes (Supplementary Tables 3 and 4). However, preterm infants were less like to have anomaly-specific admissions (aOR: 0.47; 0.42-0.52), whereas infants with mothers having a history of anomalies in previous pregnancies were more likely to have anomaly-specific admissions (aOR: 1.26; 1.11-1.43).

Discussion

Using linked de-identified data from CARIS, PEDW and ONS, we investigated a comprehensive range of risk factors associated with hospital admissions during infancy in babies born with congenital anomalies or rare diseases of congenital origin. Preterm birth was the strongest risk factor associated with an increased risk of one and two or more infant hospitalisation across all anomaly or rare disease of congenital origin subgroups. In addition, infants with two or more admissions had a higher risk profile than those with only one or no admission. Infants who had two or more admissions were more likely to be boys, born preterm, or to have mothers who were younger and smokers or for those who were born with a moderately severe CHD anomaly, compared to those with one or no admission.

Previous studies have shown that infants with a congenital anomaly were associated with a significantly high risk of hospitalisation compared to infants without an anomaly in the general population due to anomaly-related comorbidities and

Confidential: For Review Only

complications, which require ongoing inpatient treatment and investigations [6, 20-22].

Maternal and infant factors have an impact on hospitalisation in infant with congenital anomalies through different potential mechanisms. For example, previous studies have shown that teenage mothers may have an increased risk of adverse neonatal outcomes due to inadequate antenatal care and social support, and other behavioural determinants such as maternal smoking, alcohol and recreational drug misuse compared to older mothers [23]. Infants born to older mothers tend to have a reduced risk of unintentional injuries, an increased uptake of routine immunisation in childhood and may receive better parenting [24]. It has been shown that maternal smoking during pregnancy increases the risk of respiratory and infectious diseases in infants [25-27]; possible mechanisms include intrauterine stress and impaired immune system due to nicotine substances and tobacco smoking, although the exact biological mechanism is not clearly defined [28]. In our subgroup analysis, we found no significant difference in the relationship between maternal age and anomaly-specific admissions or other-cause admissions, or those between maternal smoking and anomaly-specific admissions or other-cause admissions, which suggests that the mechanisms by which maternal age and smoking are risk factors for hospitalisation of infants with an anomaly are similar for anomaly-specific admissions and admissions due to other causes.

Preterm birth has consistently been shown to be associated with an increased risk of infant morbidity and hospitalisation; preterm infants generally have a lower physiological reserve and a higher susceptibility to comorbidities and infections

Confidential: For Review Only

compared to term infants [29]. In addition, clinicians may be more cautious and adopt a lower threshold for admitting preterm infants with a congenital anomaly when complications arise [30]. Previous twin studies have shown that boys have an increased risk of respiratory distress syndrome, intrauterine growth restriction and overall morbidities compared with girls and therefore are more likely to require hospitalisation [16, 31-33]. Our subgroup analysis suggests that many infants with congenital anomalies who were born preterm required hospitalisation for conditions related to prematurity rather than the congenital anomaly, although it is possible that clinicians tend to ascribe 'prematurity' as the primary cause of admission for preterm babies regardless of their anomaly status.

Infants born with the most severe CHD (e.g. single ventricle and hypoplastic heart syndromes) often experience life-threatening events at birth and hence the mortality risk among these infants is considerably higher during their first admission compared to infants with less severe CHD [34, 35]. On the other hand, infants born with a moderately severe CHD are now more likely to survive due to advances in diagnostics, and medical and surgical interventions [36]; however, they are also likely to experience long-term morbidities and complications [5, 37, 38], which may explain the highest risk of repeated hospitalisation in this group of infants.

The risk factors for increased hospitalisation in infants with congenital anomalies found in this study are similar to known risk factors for hospitalisation in the general paediatric population, in which previous studies have found that being a boy and maternal smoking are associated with preterm birth leading to increased risk of hospitalisation [39, 40] Having a major anomaly increases the chance of an infant

Confidential: For Review Only

being born preterm [41, 42], and they may contribute to the same chain of event leading to hospitalisation.

The main strengths of this study include the national study population, robust study design and data collection. Infants with congenital anomalies or rare diseases of congenital origin were identified from CARIS, an active surveillance and multiple source reporting system covering all livebirths in Wales which maximises case finding and internal validity. The use of routinely collected data from PEDW is particularly advantageous for this hospitalisation study due to the large size of the dataset and long period of data collection. A further strength relates to the broad definition of congenital anomalies, which includes structural and chromosomal defects, as well as rare diseases of congenital origin such as metabolic, endocrine and congenital blood disorders. Consequently, we were able to investigate risk factors associated with hospitalisation of infants with a wide range of congenital anomalies and rare diseases of congenital origin.

There are several limitations. As PEDW is a hospital administrative dataset, data on admissions was not always complete which can be due to infants being treated in a specialist centre outside the catchment area of NHS Wales. In addition, the number of variables explored in this study was restricted by what was available in the dataset. It is possible that institutional factors such as outpatient follow ups which were not available in this dataset are also important determinants of hospitalisation of infants with congenital anomalies.

Confidential: For Review Only

Conclusions

Infants with congenital anomalies and rare diseases of congenital origin have a high risk of hospitalisation in infancy. Our findings can help clinicians to identify infants who are likely to require hospitalisation according to their socio-demographic and clinical characteristics. Public health interventions such as promoting good prenatal care and smoking cessation may have an important role in reducing the need for hospitalisation in infants with a congenital anomaly.

Confidential: For Review Only

What is known about the subject:

- Congenital anomalies are a leading cause of infant morbidity and hospitalisation.
- Evidence about the risk factors contributing to the hospitalisation of infants with congenital anomalies is limited.

What this study adds

- Infants who had ≥2 admissions were more likely to be boys, born preterm, born with a moderately severe CHD, or have mothers who were younger and smokers, compared to those with ≤1 admission.
- Preterm birth is the strongest risk factor for hospitalisation in infants with congenital anomalies or rare diseases of congenital origin.
- The mechanisms by which maternal age, smoking and infant sex are risk factors for hospitalisation are similar for anomaly-specific admissions and admissions due to other causes.

Confidential: For Review Only

Acknowledgements

We thank the SAIL team for providing the datasets and their assistance with data linkage for this study, and the CARIS team for their support on the dataset.

Funding

This study is part-funded by the National Institute for Health Research (NIHR) Policy Research Programme, conducted through the NIHR Policy Research Unit in Maternal Health and Care, 108/0001. The views expressed are those of the author(s) and not necessarily those of the NIHR or the Department of Health and Social Care.

Peter Ho is funded by a Clarendon – Green Templeton College – Nuffield Department of Population Health Scholarship, University of Oxford.

Competing interests

None.

Confidential: For Review Only

Contributorship statement:

PH contributed to the design of the study, acquisition, analysis, interpretation of data, drafting and revising it critically, approved the final version to be published, and agrees to be accountable for all aspects of the work in ensuring that questions related to the accuracy or integrity of any part of the work are appropriately investigated and resolved.

MQ and JK contributed to the design of the study, acquisition, analysis, and interpretation of data for the work; supervised the project and revised it critically; approved the final version to be published; and agree to be accountable for all aspects of the work in ensuring that questions related to the accuracy or integrity of any part of the work are appropriately investigated and resolved.

DT contributed to the acquisition, interpretation of data for the study, revised it critically, approved the final version to be published, and agrees to be accountable for all aspects of the work in ensuring that questions related to the accuracy or integrity of any part of the work are appropriately investigated and resolved.

For Review Only

Table 1: Factors associated with hospitalisation of infants with any anomaly or rare disease of congenital origin.

Characteristics	Number of admissions		
	2+<br>No. of infants (%)<br>*n = 10,785*	1<br>No. of infants (%)<br>*n = 6,043*	0<br>No. of infants (%)<br>*n = 8,695*
Socio-demographic factors			
Townsend quintile			
1 (least deprived)	1,542 (14.3)	991 (16.4)	1,272 (16.3)
2	1,645 (15.3)	956 (15.8)	1,316 (16.9)
3	1,928 (17.9)	1,102 (18.3)	1,571 (20.1)
4	2,351 (21.8)	1,257 (20.8)	1,558 (20.0)
5 (most deprived)	3,296 (30.6)	1,727 (28.6)	2,080 (26.7)
Maternal ethnicity			
White	6,729 (94.5)	3,547 (93.8)	4,252 (95.0)
Other	394 (5.5)	236 (6.2)	224 (5.0)
Maternal age at birth, years			
Mean (SD) (year)	*27.8 (6.3)*	*28.3 (6.2)*	*28.6 (6.2)*
Median (IQR)	*28 (23-32)*	*28 (24-33)*	*29 (24-33)*
≤ 24	3,596 (33.3)	1,768 (29.3)	2,204 (28.2)
25 – 29	2,880 (26.7)	1,697 (28.1)	2,096 (26.8)
30 – 34	2,585 (24.0)	1,526 (25.3)	2,046 (26.2)
≥ 35	1,720 (16.0)	1,050 (17.4)	1,468 (18.9)
Maternal factors			
Parity			
Nulliparous	4,572 (45.2)	2,454 (43.6)	3,029 (43.3)
≥ 1	5,535 (54.8)	3,173 (56.4)	3,973 (56.7)
Multiple fetus			
Yes	417 (3.9)	175 (2.9)	185 (2.4)
No	10,365 (96.1)	5,866 (97.1)	7,640 (97.6)
Maternal smoking			
Smoker	2,200 (30.7)	1,036 (27.1)	1,136 (25.5)
Non/ Ex smoker	4,958 (69.3)	2,783 (72.9)	3,315 (74.5)
Anomalies in previous pregnancies			
Yes	928 (12.0)	506 (12.2)	582 (12.0)
No	6,811 (88.0)	3,637 (87.8)	4,280 (88.0)
Infant factors			
Infant sex			
Male	6,595 (61.2)	3,599 (59.6)	5,205 (59.9)
Female	4,187 (38.8)	2,441 (40.4)	3,486 (40.1)
Birthweight, grams			
Mean (SD)	*3,024 (810)*	*3,207 (697)*	*3,286 (660)*
Median (IQR)	*3,140 (2,610-3,570)*	*3,280 (2,860-3,660)*	*3,350 (2,950-3,710)*
< 2500 (low birthweight)	2,311 (21.5)	815 (13.5)	806 (10.1)
≥ 2500	8,441 (78.5)	5,209 (86.4)	7,166 (89.9)
Gestational age, week			
Mean (SD)	*37.9 (3.3)*	38.6 (2.6)	39.0 (2.3)
Median (IQR)	*39 (37-40)*	39 (38-40)	39 (38-40)
< 37 (preterm)	2,287 (21.3)	844 (14.0)	727 (9.1)
≥ 37 (term)	8,465 (78.7)	5,178 (86.0)	7,249 (90.9)
Note: Missing data are not shown due to risk of statistical disclosure.			

Confidential: For Review Only

Table 2: Univariable analyses: factors associated with repeated (2+) and single (1) admissions by anomaly/rare disease subgroup.

	2+ admissions			1 admission		
	Unadjusted RRR[2]			Unadjusted RRR[2]		
Factors	All	Isolated	Multiple	All	Isolated	Multiple
Townsend quintile						
1	1 (reference)	1 (reference)	1 (reference)	1 (reference)	1 (reference)	1 (reference)
2	1.03 (0.93-1.14)	1.04 (0.92-1.18)	1.06 (0.88-1.28)	0.93 (0.83-1.05)	0.88 (0.77-1.01)	1.09 (0.87-1.37)
3	1.01 (0.92-1.12)	1.06 (0.93-1.20)	0.93 (0.78-1.10)	0.90 (0.81-1.01)	0.88 (0.77-1.01)	0.92 (0.74-1.14)
4	1.24 (1.13-1.37)	1.23 (1.08-1.39)	1.24 (1.04-1.47)	1.04 (0.93-1.16)	1.02 (0.89-1.16)	1.08 (0.87-1.34)
5	1.31 (1.19-1.43)	1.32 (1.18-1.48)	1.28 (1.08-1.51)	1.07 (0.96-1.18)	1.02 (0.90-1.15)	1.17 (0.96-1.44)
Maternal ethnicity						
White	1 (reference)	1 (reference)	1 (reference)	1 (reference)	1 (reference)	1 (reference)
Other	1.11 (0.94-1.32)	1.10 (0.88-1.38)	1.06 (0.82-1.39)	1.26 (1.05-1.52)	1.34 (1.06-1.69)	1.13 (0.82-1.56)
Maternal age at delivery, years						
≤ 24	1.19 (1.10-1.28)	1.16 (1.05-1.27)	1.23 (1.07-1.41)	0.99 (0.91-1.09)	0.97 (0.87-1.07)	1.05 (0.88-1.25)
25 – 29	1 (reference)	1 (reference)	1 (reference)	1 (reference)	1 (reference)	1 (reference)
30 – 34	0.92 (0.85-1.00)	0.89 (0.81-0.98)	0.94 (0.81-1.09)	0.92 (0.84-1.01)	0.93 (0.83-1.04)	0.90 (0.75-1.07)
≥ 35	0.53 (0.49-0.58)	0.50 (0.45-0.56)	0.55 (0.48-0.64)	0.55 (0.50-0.61)	0.54 (0.48-0.61)	0.58 (0.48-0.69)
Parity						
Nulliparous	1 (reference)	1 (reference)	1 (reference)	1 (reference)	1 (reference)	1 (reference)
≥ 1	0.73 (0.69-0.77)	0.73 (0.68-0.79)	0.76 (0.68-0.84)	0.78 (0.73-0.84)	0.78 (0.72-0.85)	0.80 (0.71-0.91)
Multiple fetus						
Yes	1.66 (1.39-1.98)	1.92 (1.53-2.39)	1.20 (0.89-1.60)	1.23 (1.00-1.52)	1.43 (1.11-1.84)	0.87 (0.60-1.27)
No	1 (reference)	1 (reference)	1 (reference)	1 (reference)	1 (reference)	1 (reference)
Maternal smoking						
Smoker	1.30 (1.19-1.41)	1.32 (1.19-1.48)	1.24 (1.09-1.43)	1.09 (0.99-1.20)	1.06 (0.94-1.20)	1.14 (0.96-1.34)
Non/Ex smoker	1 (reference)	1 (reference)	1 (reference)	1 (reference)	1 (reference)	1 (reference)
Anomalies in previous pregnancies						
Yes	1.00 (0.89-1.11)	1.04 (0.91-1.20)	0.81 (0.67-0.99)	0.98 (0.86-1.11)	1.00 (0.86-1.16)	0.90 (0.70-1.14)
No	1 (reference)	1 (reference)	1 (reference)	1 (reference)	1 (reference)	1 (reference)
Infant sex[1]						
Male	1 (reference)	1 (reference)	1 (reference)	1 (reference)	1 (reference)	1 (reference)
Female	0.95 (0.90-1.00)	0.94 (0.87-1.01)	0.88 (0.79-0.97)	1.01 (0.95-1.08)	1.07 (0.99-1.16)	0.86 (0.76-0.97)
Gestational age[1], week						
< 37	2.95 (2.70-3.23)	3.25 (2.89-3.64)	2.15 (1.87-2.49)	1.79 (1.61-1.98)	1.91 (1.67-2.18)	1.48 (1.24-1.77)
≥ 37	1 (reference)	1 (reference)	1 (reference)	1 (reference)	1 (reference)	1 (reference)

[1]Variable identified for inclusion a priori. [2]Statistically significant at $p < 0.1$ for unadjusted RRR. Note: RRR is relative to the risk in the zero admission patients.

Confidential: For Review Only

Table 3: Multivariable analyses: factors associated with repeated (2+) and single (1) admissions by anomaly or rare disease of congenital origin subgroup.

	2+ admissions			1 admission		
	Adjusted RRR[2]			Adjusted RRR[2]		
Factors	All	Isolated	Multiple	All	Isolated	Multiple
Maternal age at delivery, years						
≤ 24	1.17 (1.06-1.30)		1.22 (1.06-1.41)	1.00 (0.89-1.12)		1.04 (0.88-1.24)
25 – 29	1 (reference)		1 (reference)	1 (reference)		1 (reference)
30 – 34	0.99 (0.89-1.10)		0.94 (0.81-1.09)	0.94 (0.83-1.06)		0.90 (0.75-1.07)
≥ 35	0.93 (0.82-1.04)		0.54 (0.47-0.63)	0.95 (0.83-1.09)		0.58 (0.48-0.69)
Maternal smoking						
Smoker	1.20 (1.10-1.31)	1.26 (1.13-1.41)		1.06 (0.96-1.17)	1.04 (0.92-1.18)	
Non/Ex smoker	1 (reference)	1 (reference)		1 (reference)	1 (reference)	
Infant sex[1]						
Male	1 (reference)	1 (reference)	1 (reference)	1 (reference)	1 (reference)	1 (reference)
Female	0.84 (0.78-0.91)	0.82 (0.74-0.90)	0.90 (0.81-1.00)	0.95 (0.87-1.04)	1.01 (0.91-1.12)	0.87 (0.77-0.99)
Gestational age[1], week						
< 37	2.52 (2.25-2.83)	2.83 (2.41-3.31)	2.19 (1.90-2.53)	1.68 (1.47-1.92)	1.85 (1.55-2.21)	1.50 (1.26-1.79)
≥ 37	1 (reference)	1 (reference)	1 (reference)	1 (reference)	1 (reference)	1 (reference)

[1]*Variable identified for inclusion a priori.*
[2]*For 'All' anomaly subgroup, adjusted variables include maternal age at delivery, maternal smoking, infant sex and gestational age; for 'isolate' anomaly subgroup, adjusted variables include maternal smoking, infant sex and gestation age; for 'multiple' anomaly subgroup, adjusted variables include maternal age at delivery, infant sex and gestational age. Statistical significance at $p < 0.05$.*

Confidential: For Review Only

Table 4: Unadjusted and adjusted factors associated with repeated (2+) and single (1) admissions of infants with cardiovascular anomalies.

	2+ admissions		1 admission	
	Cardiovascular, *n = 997*			
Factors	Unadjusted RRR[2]	Adjusted RRR[3]	Unadjusted RRR[2]	Adjusted RRR[3]
Townsend quintile				
1	1 (reference)		1 (reference)	
2	1.11 (0.80-1.53)		0.80 (0.56-1.12)	
3	1.35 (0.99-1.85)		0.94 (0.68-1.31)	
4	1.10 (0.81-1.50)		1.05 (0.77-1.43)	
5	1.40 (1.05-1.85)		0.97 (0.72-1.30)	
Maternal ethnicity				
White	1 (reference)		1 (reference)	
Other	0.82 (0.49-1.38)		0.91 (0.53-1.57)	
Maternal age at delivery, years				
≤ 24	1.20 (0.95-1.53)		0.94 (0.72-1.23)	
25 – 29	1 (reference)		1 (reference)	
30 – 34	1.02 (0.80-1.31)		1.08 (0.83-1.41)	
≥ 35	0.57 (0.43-0.75)		0.67 (0.51-0.90)	
Parity				
Nulliparous	1 (reference)		1 (reference)	
≥ 1	0.92 (0.76-1.11)		0.86 (0.70-1.05)	
Multiple fetus				
Yes	1.86 (1.15-3.00)		1.55 (0.91-2.62)	
No	1 (reference)		1 (reference)	
Maternal smoking				
Smoker	1.39 (1.07-1.79)		1.07 (0.81-1.42)	
Non/Ex smoker	1 (reference)		1 (reference)	
Anomalies in previous pregnancies				
Yes	1.32 (0.94-1.86)		1.20 (0.84-1.71)	
No	1 (reference)		1 (reference)	
Infant sex[1]				
Male	1 (reference)	1 (reference)	1 (reference)	1 (reference)
Female	0.66 (0.55-0.79)	0.75 (0.62-0.91)	0.95 (0.78-1.15)	0.95 (0.78-1.15)
Gestational age[1], week				
< 37	3.33 (2.55-4.35)	3.76 (2.86-4.93)	1.97 (1.46-2.65)	1.97 (1.46-2.66)
≥ 37	1 (reference)	1 (reference)	1 (reference)	1 (reference)
CHD Disease Severity[1]				
Less severity	1 (reference)	1 (reference)	1 (reference)	1 (reference)
Moderate severity	5.83 (4.19-8.11)	6.25 (4.47-8.74)	1.14 (0.75-1.75)	1.18 (0.77-1.81)
Most severe	2.67 (1.57-4.57)	2.72 (1.58-4.71)	0.38 (0.15-0.94)	0.39 (0.16-0.96)

[1]Variable identified for inclusion a priori. [2]Statistically significant at $p < 0.1$ for the unadjusted RRR. [3]Adjusted variables include infant sex, gestational age and disease severity. Statistically significant at $p < 0.05$.

Confidential: For Review Only

References

1. Yoon, P.W., Olney, R.S., Khoury, M.J., Sappenfield, W.M., Chavez, G.F. and Taylor, D., 1997. Contribution of birth defects and genetic diseases to pediatric hospitalizations: a population-based study. *Archives of pediatrics & adolescent medicine*, *151*(11), pp.1096-1103.
2. Cherian, J., Staggers, K.A., Pan, I.W., Lopresti, M., Jea, A. and Lam, S., 2016. Thirty-day outcomes after postnatal myelomeningocele repair: a National Surgical Quality Improvement Program Pediatric database analysis. *Journal of Neurosurgery: Pediatrics*, *18*(4), pp.416-422.
3. Bradnock, T.J., Marven, S., Owen, A., Johnson, P., Kurinczuk, J.J., Spark, P., Draper, E.S. and Knight, M., 2011. Gastroschisis: one year outcomes from national cohort study. *Bmj*, *343*, p.d6749.
4. Derrington, T.M., Kotelchuck, M., Plummer, K., Cabral, H., Lin, A.E., Belanoff, C., Shin, M., Correa, A. and Grosse, S.D., 2013. Racial/ethnic differences in hospital use and cost among a statewide population of children with Down syndrome. *Research in developmental disabilities*, *34*(10), pp.3276-3287.
5. Marston, A.P., Patel, T., Nguyen, S.A. and White, D.R., 2019. Short-Term Risk Factor Profile of Pediatric Choanal Atresia Repair Using ACS-NSQIP National Database. *Annals of Otology, Rhinology & Laryngology*, *128*(9), pp.855-861.
6. Medrano, C., Garcia-Guereta, L., Grueso, J., Insa, B., Ballesteros, F., Casaldaliga, J., Cuenca, V., Escudero, F., de la Calzada, L.G., Luis, M. and Luque, M., 2007. Respiratory infection in congenital cardiac disease. Hospitalizations in young children in Spain during 2004 and 2005: the CIVIC Epidemiologic Study. *Cardiology in the Young*, *17*(4), pp.360-371.
7. Fitzsimons, K.J., Copley, L.P., Deacon, S.A. and van der Meulen, J.H., 2013. Hospital care of children with a cleft in England. *Archives of disease in childhood*, *98*(12), pp.970-974.
8. Razzaghi, H., Dawson, A., Grosse, S.D., Allori, A.C., Kirby, R.S., Olney, R.S., Correia, J. and Cassell, C.H., 2015. Factors associated with high hospital resource use in a population-based study of children with orofacial clefts. *Birth Defects Research Part A: Clinical and Molecular Teratology*, *103*(2), pp.127-143.

https://mc.manuscriptcentral.com/bmjpo

9. Congenital Anomaly Register and Information Service (CARIS) 2018, *Introduction. Congenital Anomaly Register and information Service*, CARIS, viewed 8 March 2019, <http://www.caris.wales.nhs.uk/introduction>.

10. National Health Service (NHS) Wales Information Service 2020, *PEDW Data Online*, NHS Wales Information Service, viewed 28 December 2019, <http://www.infoandstats.wales.nhs.uk/page.cfm?orgid=869&pid=40977>.

11. Office for National Statistics (ONS) 2015, *Statistical bulletin: Birth Summary Tables, England and Wales: 2014,* ONS, viewed 8 September 2019, <https://www.ons.gov.uk/peoplepopulationandcommunity/birthsdeathsandmarriages/livebirths/bulletins/birthsummarytablesenglandandwales/2015-07-15>.

12. NHS Wales Digital Health and Care Wales, 2021, *NHS Wales Data Dictionary,* NHS Wales Digital Health and Care Wales, viewed 23 May 2021, <http://www.datadictionary.wales.nhs.uk/>.

13. European Surveillance of Congenital Anomalies (EUROCAT) 2013, EUROCAT Guide 1.4: Instruction for the registration of congenital anomalies. EUROCAT Central Registry, University of Ulster. Last update version 28/12/2018, viewed 25 August 2019, <https://eu-rd-platform.jrc.ec.europa.eu/sites/default/files/Full_Guide_1_4_version_28_DEC2018.pdf>.

14. World Health Organization (WHO), 2004. ICD-10: international statistical classification of diseases and related health problems: tenth revision.

15. Butler, A.S. and Behrman, R.E. eds., 2007. *Preterm birth: causes, consequences, and prevention*. National academies press.

16. Stevenson, D.K., Verter, J., Fanaroff, A.A., Oh, W., Ehrenkranz, R.A., Shankaran, S., Donovan, E.F., Wright, L.L., Lemons, J.A., Tyson, J.E. and Korones, S.B., 2000. Sex differences in outcomes of very low birthweight infants: the newborn male disadvantage. *Archives of disease in childhood-fetal and neonatal edition*, *83*(3), pp.F182-F185.

17. Islam, S., Yasui, Y., Kaul, P., Marelli, A.J. and Mackie, A.S., 2016. Congenital heart disease hospitalizations in Canada: a 10-year experience. *Canadian Journal of Cardiology*, *32*(2), pp.197-203.

18. Mackie, A.S., Tran, D.T., Marelli, A.J. and Kaul, P., 2017. Cost of congenital heart disease hospitalizations in Canada: a population-based study. *Canadian Journal of Cardiology*, *33*(6), pp.792-798.

19. StataCorp. 2013. *Stata Statistical Software: Release 13*. College Station, TX: StataCorp LP.

20. Colvin, L. and Bower, C., 2009. A retrospective population-based study of childhood hospital admissions with record linkage to a birth defects registry. *BMC pediatrics*, *9*(1), p.32.

21. Wynn, J., Krishnan, U., Aspelund, G., Zhang, Y., Duong, J., Stolar, C.J., Hahn, E., Pietsch, J., Chung, D., Moore, D. and Austin, E., 2013. Outcomes of congenital diaphragmatic hernia in the modern era of management. *The Journal of pediatrics*, *163*(1), pp.114-119.

22. Frid, C., Annerén, G., Rasmussen, F., Sundelin, C. and Drott, P., 2002. Utilization of medical care among children with Down's syndrome. *Journal of Intellectual Disability Research*, *46*(4), pp.310-317.

23. Harper, L.M., Chang, J.J. and Macones, G.A., 2011. Adolescent pregnancy and gestational weight gain: do the Institute of Medicine recommendations apply?. *American journal of obstetrics and gynecology*, *205*(2), pp.140-e1.

24. Sutcliffe, A.G., Barnes, J., Belsky, J., Gardiner, J. and Melhuish, E., 2012. The health and development of children born to older mothers in the United Kingdom: observational study using longitudinal cohort data. *Bmj*, *345*, p.e5116.

25. DiFranza, J.R., Aligne, C.A. and Weitzman, M., 2004. Prenatal and postnatal environmental tobacco smoke exposure and children's health. *Pediatrics*, *113*(Supplement 3), pp.1007-1015.

26. Håberg, S.E., Stigum, H., Nystad, W. and Nafstad, P., 2007. Effects of pre-and postnatal exposure to parental smoking on early childhood respiratory health. *American journal of epidemiology*, *166*(6), pp.679-686.

27. Yusuf, H.R., Rochat, R.W., Baughman, W.S., Gargiullo, P.M., Perkins, B.A., Brantley, M.D. and Stephens, D.S., 1999. Maternal cigarette smoking and invasive meningococcal disease: a cohort study among young children in metropolitan Atlanta, 1989-1996. *American journal of public health*, *89*(5), pp.712-717.

Confidential: For Review Only

28. Weinsheimer, R.L. and Yanchar, N.L., 2008. Impact of maternal substance abuse and smoking on children with gastroschisis. *Journal of pediatric surgery*, *43*(5), pp.879-883.
29. Björ, O. and Bråbäck, L., 2003. A retrospective population based trend analysis on hospital admissions for lower respiratory illness among Swedish children from 1987 to 2000. *BMC Public Health*, *3*(1), p.22.
30. Mackie, A.S., Ionescu-Ittu, R., Pilote, L., Rahme, E. and Marelli, A.J., 2008. Hospital readmissions in children with congenital heart disease: a population-based study. *American heart journal*, *155*(3), pp.577-584.
31. Lisi, A., Botto, L.D., Rittler, M., Castilla, E., Bianchi, F., Botting, B., De Walle, H., Erickson, J.D., Gatt, M., De Vigan, C. and Irgens, L., 2005. Sex and congenital malformations: an international perspective. *American Journal of Medical Genetics Part A*, *134*(1), pp.49-57.
32. Mulla, Z.D., Plavsic, S.K., Ortiz, M., Nuwayhid, B.S. and Ananth, C.V., 2013. Fetal sex pairing and adverse perinatal outcomes in twin gestations. *Annals of epidemiology*, *23*(1), pp.7-12.
33. Kent, A.L., Wright, I.M. and Abdel-Latif, M.E., 2012. Mortality and adverse neurologic outcomes are greater in preterm male infants. *Pediatrics*, *129*(1), pp.124-131.
34. van Dongen, E.I., Glansdorp, A.G., Mildner, R.J., McCrindle, B.W., Sakopoulos, A.G., VanArsdell, G., Williams, W.G. and Bohn, D., 2003. The influence of perioperative factors on outcomes in children aged less than 18 months after repair of tetralogy of Fallot. *The Journal of thoracic and cardiovascular surgery*, *126*(3), pp.703-710.
35. Desai, J., Aggarwal, S., Lipshultz, S., Agarwal, P., Yigazu, P., Patel, R., Seals, S. and Natarajan, G., 2017. Surgical Interventions in Infants Born Preterm with Congenital Heart Defects: An Analysis of the Kids' Inpatient Database. *The Journal of pediatrics*, *191*, pp.103-109.
36. Holst, K.A., Said, S.M., Nelson, T.J., Cannon, B.C. and Dearani, J.A., 2017. Current interventional and surgical management of congenital heart disease: specific focus on valvular disease and cardiac arrhythmias. *Circulation research*, *120*(6), pp.1027-1044.
37. Smith, A.H., Doyle, T.P., Mettler, B.A., Bichell, D.P. and Gay, J.C., 2015. Identifying predictors of hospital readmission following congenital heart surgery through

Confidential: For Review Only

analysis of a multiinstitutional administrative database. *Congenital heart disease*, *10*(2), pp.142-152.

38. Duppenthaler, A., Ammann, R.A., Gorgievski-Hrisoho, M., Pfammatter, J.P. and Aebi, C., 2004. Low incidence of respiratory syncytial virus hospitalisations in haemodynamically significant congenital heart disease. *Archives of disease in childhood*, *89*(10), pp.961-965.
39. Stock, S.J. and Bauld, L., 2020. Maternal smoking and preterm birth: An unresolved health challenge. *PLoS Medicine*, *17*(9), p.e1003386.
40. Zeitlin, J., Saurel-Cubizolles, M.J., De Mouzon, J., Rivera, L., Ancel, P.Y., Blondel, B. and Kaminski, M., 2002. Fetal sex and preterm birth: are males at greater risk?. *Human reproduction*, *17*(10), pp.2762-2768.
41. Butler, A.S. and Behrman, R.E. eds., 2007. *Preterm birth: causes, consequences, and prevention*. National academies press.
42. Dolan, S.M., Gross, S.J., Merkatz, I.R., Faber, V., Sullivan, L.M., Malone, F.D., Porter, T.F., Nyberg, D.A., Comstock, C.H., Hankins, G.D. and Eddleman, K., 2007. The contribution of birth defects to preterm birth and low birth weight. *Obstetrics & Gynecology*, *110*(2), pp.318-324.

Confidential: For Review Only

Supplementary Table 2: Year of birth added as a covariate in the final model as a sensitivity analysis.

	2+ admissions				1 admission			
	Adjusted RRR				Adjusted RRR			
Factors	All	Isolated	Multiple	Cardiovascular	All	Isolated	Multiple	Cardiovascular
Maternal age at delivery, years								
≤ 24	1.18 (1.06-1.30)		1.22 (1.06-1.41)		1.00 (0.89-1.12)		1.04 (0.88-1.24)	
25 – 29	1 (reference)		1 (reference)		1 (reference)		1 (reference)	
30 – 34	0.99 (0.89-1.10)		0.94 (0.81-1.09)		0.94 (0.83-1.06)		0.90 (0.75-1.07)	
≥ 35	0.93 (0.82-1.04)		0.54 (0.47-0.63)		0.95 (0.84-1.09)		0.58 (0.48-0.69)	
Maternal smoking								
Smoker	1.20 (1.10-1.31)	1.26 (1.13-1.41)			1.06 (0.96-1.17)	1.04 (0.92-1.18)		
Non/Ex smoker	1 (reference)	1 (reference)			1 (reference)	1 (reference)		
Infant sex[1]								
Male	1 (reference)	1 (reference)	1 (reference)	1 (reference)	1 (reference)	1 (reference)	1 (reference)	1 (reference)
Female	0.84 (0.78-0.91)	0.82 (0.74-0.90)	0.90 (0.81-1.00)	0.75 (0.62-0.91)	0.95 (0.87-1.04)	1.01 (0.91-1.12)	0.87 (0.77-0.99)	0.95 (0.78-1.15)
Gestational age[1], week								
< 37	2.52 (2.25-2.83)	2.83 (2.41-3.31)	2.19 (1.90-2.53)	3.74 (2.84-4.91)	1.68 (1.47-1.92)	1.85 (1.55-2.21)	1.50 (1.26-1.79)	1.97 (1.45-2.64)
≥ 37	1 (reference)	1 (reference)	1 (reference)	1 (reference)	1 (reference)	1 (reference)	1 (reference)	1 (reference)
CHD Disease Severity[1]								
Less severity	N/A	N/A	N/A	1 (reference)	N/A	N/A	N/A	1 (reference)
Moderate severity				6.31 (4.51-8.83)				1.20 (0.78-1.84)
Most severe				2.76 (1.60-4.77)				0.39 (0.16-0.98)
For each year increment between 1999 and 2016.	1.00 (0.99-1.01)	1.00 (0.99-1.01)	1.00 (0.99-1.01)	0.99 (0.97-1.01)	0.99 (0.98-1.00)	0.99 (0.97-1.00)	1.00 (0.98-1.01)	0.98 (0.96-1.00)

[1]*Variable identified for inclusion a priori. N/A = not applicable.*

Confidential: For Review Only

Supplementary Table 1: Inclusion of anomaly and rare disease of congenital origin subgroups.

Subgroup	Classification of anomaly and rare disease of congenital origin included
All anomalies; all isolated anomalies; and all multiple anomalies	• Nervous system;<br>• Eye, ear, face and neck;<br>• Cardiovascular system;<br>• Respiratory and clefts;<br>• Abdominal wall defects and diaphragmatic hernia;<br>• Upper and lower gastrointestinal;<br>• Genitourinary;<br>• Limb and skeletal;<br>• Blood disorders;<br>• Endocrine and Metabolic;<br>• Neoplasm;<br>• Syndromes and congenital malformation syndromes;<br>• Maternal Infection leading to malformations.
Cardiovascular anomalies	• Congenital heart defects and diseases of the circulatory system.

Confidential: For Review Only

Supplementary Table 3: Factors associated with cause-specific admissions among infants with any anomaly or rare disease of congenital origin.

	Anomaly-specific admissions, no. of infants (%)	Other-cause admissions, no. of infants (%)	Unadjusted OR (95% CI)	P-value[1]
Socio-demographic factors				
Townsend quintile				
1 (least deprived)	772 (15.4)	1,709 (14.9)	1 (reference)	0.253
2	767 (15.3)	1,780 (15.6)	0.95 (0.85-1.08)	
3	932 (18.6)	2,042 (17.9)	1.01 (0.90-1.13)	
4	1,093 (21.8)	2,432 (21.3)	0.99 (0.89-1.11)	
5 (most deprived)	1,439 (28.8)	3,472 (30.4)	0.92 (0.83-1.02)	
Maternal ethnicity				
White	3,101 (94.2)	6,948 (94.2)	1 (reference)	0.978
Other	191 (5.8)	429 (5.8)	1.00 (0.84-1.19)	
Maternal age at birth, years *(n = 29,400)*				
≤ 24	1,570 (31.3)	3,680 (32.1)	0.97 (0.89-1.06)	0.712
25 – 29	1,363 (27.1)	3,103 (27.1)	1 (reference)	
30 – 34	1,251 (24.9)	2,782 (24.3)	1.02 (0.93-1.22)	
≥ 35	831 (16.6)	1,891 (16.5)	1.00 (0.90-1.11)	
Maternal factors				
Parity				
Nulliparous	2,101 (41.9)	4,782 (41.7)	1 (reference)	0.856
≥ 1	2,914 (58.1)	6,674 (58.3)	0.99 (0.93-1.06)	
Multiple fetus				
Yes	153 (3.1)	427 (3.7)	0.81 (0.67-0.98)	0.028
No	4,862 (96.9)	11,029 (96.3)	1 (reference)	
Maternal smoking				
Smoker	946 (28.0)	2,218 (30.1)	0.90 (0.83-0.99)	0.027
Non/ Ex smoker	2,430 (72.0)	5,147 (69.9)	1 (reference)	
Anomalies in previous pregnancies				
Yes	374 (10.4)	1,028 (12.8)	1.27 (1.12-1.44)	<0.001
No	3,231 (89.6)	6,981 (87.2)	1 (reference)	
Infant factors				
Infant sex				
Male	3,013 (60.1)	6,964 (60.8)	1 (reference)	0.405
Female	1,999 (39.9)	4,489 (39.2)	1.03 (0.96-1.10)	
Birthweight, grams				
< 2500	598 (11.9)	2,448 (21.4)	0.50 (0.45-0.55)	<0.0001
≥ 2500	4,417 (88.0)	9,008 (78.6)	1 (reference)	
Gestational age, week				
$< 37^{+0}$	577 (11.5)	2,470 (21.6)	0.47 (0.43-0.52)	<0.0001
$\geq 37^{+0}$	4,438 (88.5)	8,986 (78.4)	1 (reference)	

[1]P < 0.1 was considered as statistically significant. Missing data are not shown due to risk of statistical disclosure.

Confidential: For Review Only

Supplementary Table 4: Factors associated with cause-specific admissions among infants with any anomaly or rare disease of congenital origin: multivariable analysis.

Risk factors	Crude OR (95% CI)	P-value[1]	aOR[2] (95% CI)	P-value[3]
Anomalies in previous pregnancies				
Yes	1.27 (1.12-1.44)	<0.001	1.26 (1.11-1.43)	<0.001
No	1 (reference)		1 (reference)	
Gestational age, week				
< 37 (preterm)	0.47 (0.43-0.52)	<0.0001	0.47 (0.42-0.52)	<0.0001
≥ 37 (term)	1 (reference)		1 (reference)	

[1]P-value of the univariable analysis < 0.1 was considered as statistically significant. [2]Mutually adjusted odds ratios for other factors in the table. [3]P-value of the multivariable analysis < 0.05 was considered as statistically significant.

Confidential: For Review Only

BMJ Paediatrics Open

Risk factors for hospitalisation in Welsh infants with a congenital anomaly

Journal:	*BMJ Paediatrics Open*
Manuscript ID	bmjpo-2021-001238.R2
Article Type:	Original research
Date Submitted by the Author:	30-Nov-2021

Complete List of Authors:	Ho, Peter ; University of Oxford, Nuffield Department of Population Health<br>Quigley, Maria; University of Oxford, National Perinatal Epidemiology Unit, Nuffield Department of Population Health; University of Oxford, National Institute for Health Research (NIHR) Policy Research Unit-Maternal Health and Care, National Perinatal Epidemiology Unit, Nuffield Department of Population Health<br>Tucker, David; Public Health Wales, Public Health Wales, Public Health Knowledge & Research, Congenital Anomaly Register & information Service for Wales, Swansea.<br>Kurinczuk, Jenny; University of Oxford, National Perinatal Epidemiology Unit, Nuffield Department of Population Health; University of Oxford, National Institute for Health Research (NIHR) Policy Research Unit-Maternal Health and Care, National Perinatal Epidemiology Unit, Nuffield Department of Population Health

Keywords:	Epidemiology, Neonatology, Health services research

SCHOLARONE™
Manuscripts

I, the Submitting Author has the right to grant and does grant on behalf of all authors of the Work (as defined in the below author licence), an exclusive licence and/or a non-exclusive licence for contributions from authors who are: i) UK Crown employees; ii) where BMJ has agreed a CC-BY licence shall apply, and/or iii) in accordance with the terms applicable for US Federal Government officers or employees acting as part of their official duties; on a worldwide, perpetual, irrevocable, royalty-free basis to BMJ Publishing Group Ltd ("BMJ") its licensees and where the relevant Journal is co-owned by BMJ to the co-owners of the Journal, to publish the Work in this journal and any other BMJ products and to exploit all rights, as set out in our licence.

The Submitting Author accepts and understands that any supply made under these terms is made by BMJ to the Submitting Author unless you are acting as an employee on behalf of your employer or a postgraduate student of an affiliated institution which is paying any applicable article publishing charge ("APC") for Open Access articles. Where the Submitting Author wishes to make the Work available on an Open Access basis (and intends to pay the relevant APC), the terms of reuse of such Open Access shall be governed by a Creative Commons licence – details of these licences and which Creative Commons licence will apply to this Work are set out in our licence referred to above.

Other than as permitted in any relevant BMJ Author's Self Archiving Policies, I confirm this Work has not been accepted for publication elsewhere, is not being considered for publication elsewhere and does not duplicate material already published. I confirm all authors consent to publication of this Work and authorise the granting of this licence.

Confidential: For Review Only

RISK FACTORS FOR HOSPITALISATION IN WELSH INFANTS WITH A CONGENITAL ANOMALY.

Peter Ho[1], Maria A Quigley[1,2], David Tucker[3], Jennifer J Kurinczuk[1,2]

[1]National Perinatal Epidemiology Unit, Nuffield Department of Population Health, University of Oxford.

[2]National Institute for Health Research (NIHR) Policy Research Unit- Maternal Health and Care, National Perinatal Epidemiology Unit, Nuffield Department of Population Health, University of Oxford.

[3]Public Health Wales, Public Health Knowledge & Research, Congenital Anomaly Register & information Service for Wales, Swansea.

Correspondence author: Dr. Peter Ho, National Perinatal Epidemiology Unit, Nuffield Department of Population Health, University of Oxford, Old Road Campus, Headington, Oxford OX3 7LF, UK. Email: Peterhosy@hotmail.com Tel: +44 7905497825 Fax: +44 1865 289701

Word count: 2500 (excluding abstract and references)

Confidential: For Review Only

Abstract

Objectives: To investigate risk factor associated with hospitalisation of infants with a congenital anomaly in Wales, UK.

Design: A population-based cohort study.

Setting: Data from the Welsh Congenital Anomaly Register and Information Service (CARIS) linked to the Patient Episode Database for Wales (PEDW) and livebirths and deaths from the Office for National Statistics (ONS).

Patients: All livebirths between 1999 and 2015 with a diagnosis of a congenital anomaly, which was defined as a structural, metabolic, endocrine, or genetic defect, as well as rare diseases of hereditary origin.

Main outcome measures: Adjusted odd ratios (aOR) associated with 1 or 2+ hospital admissions in infancy versus no admissions were estimated for socio-demographic, maternal and infant factors using multinomial logistic regression for the subgroups of all, isolated, multiple and cardiovascular anomalies.

Results: 25,523 infants affected by congenital anomalies experienced a total of 50,705 admissions in infancy. Risk factors for ≥2 admissions were younger maternal age ≤24 years (aOR: 1.17; 95% CI: 1.06-1.30), maternal smoking (aOR: 1.20; 1.10-1.31), preterm birth (aOR: 2.52; 2.25-2.82) and moderately severe congenital heart defects (aOR: 6.25; 4.47-8.74). Girls had an overall decreased risk of 2+ admissions (aOR: 0.84; 0.78-0.91). Preterm birth was a significant risk factor for admissions in all

Confidential: For Review Only

anomaly subgroups but the effect of the other characteristics varied according to anomaly subgroup.

Conclusions: Over two thirds of infants with an anomaly are admitted to hospital during infancy. Our findings identified socio-demographic and clinical characteristics contributing to an increased risk of hospitalisation of infants with congenital anomalies.

Confidential: For Review Only

Introduction

Congenital anomalies are a leading cause of infant morbidity and hospitalisation [1]. Infants who are born with a major congenital anomaly such as a congenital heart defect (CHD), Down syndrome and congenital diaphragmatic hernia often experience anomaly-related sequelae and complications, and require more frequent hospitalisation compared to children without an anomaly.

There is some evidence that infant characteristics can influence hospitalisation of children affected by specific anomalies. For example, previous studies have shown that preterm birth, low birthweight, disease severity and co-morbidity are significantly associated with an increased risk of excess hospitalisation of infants with spina bifida, choanal atresia, congenital heart defects, orofacial clefts, congenital diaphragmatic hernia, biliary atresia, gastroschisis and Down syndrome [2-8]; while the effects of socio-demographic and maternal are less well established. Further research on risk factors for hospitalisation, particularly those associated with a wider range of congenital anomalies are needed to inform planning of healthcare and social interventions aimed at reducing infant morbidity and hospitalisation.

We aimed to investigate risk factors for hospitalisation in the first year after birth among infants with congenital anomalies.

Confidential: For Review Only

Methods

Study design and record linkage

A national population-based cohort study was established using registry data from the Congenital Anomaly Register and Information Service (CARIS) for Wales, linked to Patient Episode Database for Wales (PEDW), and births and deaths registration data from the Office for National Statistics (ONS) [9-11], and de-identified for analysis.

Deterministic and probabilistic linkage between datasets were performed using an unique Anonymised Linkage Field (ALF) encrypted code provided by NHS Wales Informatics Service, based on infant's NHS number, name, date of birth and address. All infants in the CARIS and PEDW datasets have a validated ALF and hence each infant was matched with hospital admissions using one to multiple merge; unmatched infants were treated as zero admissions. The outputs of linkage were validated by the Secure Anonymised Information Linkage (SAIL) team.

Study population

The inclusion criteria for this study were:

1. All livebirths between 1999 and 2015 with birthweight ≥500g, gestational age $\geq 22^{+0}$ weeks, with a confirmed or probable diagnosis of a congenital anomaly reported to CARIS. CARIS defines congenital anomalies as structural, metabolic, endocrine, or genetic defects, as well as rare diseases of hereditary origin present in the child or fetus at the end of pregnancy, even if not detected until

Confidential: For Review Only

after birth [9]. Therefore, the 10th revision of International Classification of Diseases and Related Health Problems (ICD-10) 'Q', 'P35' and 'P37" codes, as well as other non 'Q' ICD-10 codes of congenital anomalies and rare diseases were also included in this study.

2. All hospital admissions (i.e. emergency, elective and transfer admissions) of infants with a congenital anomaly. An admission is defined as a hospital stay of a patient using a bed on premises controlled by the one health care provider [12]. An admission is ended by a discharge, transfer to another NHS provider or death. All infants who were admitted from birth due to medical reasons were included in the analysis.

Exclusion criteria

1. Admissions solely for the birth of babies (i.e. birth hospitalisation) were excluded.

Categorisation of anomaly

Congenital anomalies were categorised according to the European Surveillance of Congenital Anomalies (EUROCAT) subgroup classification [13]; other rare diseases which are not included in the EUROCAT subgroup classification were categorised according to the ICD chapter headings [14] (Supplementary Table 1).

Infants were considered as having an isolated congenital anomaly (or disease) if a single anomaly (or disease) was diagnosed and reported to CARIS. Infants were considered as having multiple anomalies (or diseases) if more than one anomaly (or

Confidential: For Review Only

diseases) was diagnosed, either within the same body system or involving different body systems. Infants who were diagnosed with a syndrome involving more than one anomaly (or disease) were considered as having multiple anomalies (or diseases).

Outcome

The outcome was total hospital admissions for any cause in infancy (period from birth up to the first birthday) and infants were categorised as having 0, 1 or 2+ admissions.

Statistical analysis

Multinomial logistic regression was conducted to estimate the adjusted odd ratios (aOR) of hospital admission and the corresponding 95% confidence interval (CI) according to the categories of socio-demographic, maternal and infant factors (Table 1), comparing infants who had one or 2+ admissions versus those who had no admission (the comparison group). Any hospital transfers were treated as separate admissions in order to assess the full burden and utilisation of hospitalisation.

The modeling strategy aimed to identify the strongest risk factors for hospital admission. Variables included *a priori* in the multivariable models were infant sex, gestational age at birth and disease severity for cardiovascular anomalies as these are known determinants of infant morbidity and hospitalisation [5, 15-18], and these variables were included in the final model regardless of their statistical significance in the univariable analysis. The remaining variables that were associated with

Confidential: For Review Only

hospitalisation in univariable analysis (p <0.1) were also initially included in multivariable models to observe their effect size and statistical significance and they were removed from the multivariable model if no category of the variable improved the fit of the data (i.e. p <0.05). As birthweight and gestational age at birth were highly collinear (correlation coefficient r = 0.75), only gestational age at birth was included in the final model due to its greater clinical utility. All multivariable analysis was carried out separately for the following subgroups: infants with any anomaly; infants with isolated anomalies; infants with multiple anomalies; and infants with cardiovascular anomalies.

A further subgroup analysis was conducted for those babies who had at least one admission to investigate whether risk factors were different between infants with anomaly-specific admissions and those whose admissions were for 'other-causes'. Here, anomaly-specific admissions were defined as hospitalisation of infants due to 'congenital anomalies, deformations, and chromosomal abnormalities' (ICD-10 codes: Q00-Q99/ chapter XVII) as the primary reason for admission. Crude odds ratios with corresponding 95% CIs were estimated for each explanatory variable comparing these two groups of infants. Significant variables from the univariable analysis (p <0.1) were included in the multivariable analysis. P <0.05 was considered as statistically significant in all final models.

A sensitivity analysis was conducted by adding year of birth to the final model for each subgroup to investigate if the effect of the key risk factors changed.

Confidential: For Review Only

Missing data for the majority of variables were few and they are not shown due to the risk of statistical disclosure. All analyses were performed using complete case analysis. All statistical analyses were performed using Stata 13 [19].

Research ethics and statistical disclosure

The secondary anonymised health data for this study was provided by the SAIL databank in Wales. This study was approved by the SAIL Information Governance Review Panel (IGRP) and did not require separate research ethics committee review. All statistical disclosure control and checks were strictly followed according to the SAIL Databank and ONS guidelines, to ensure that individual infants were not deductively identifiable.

Patient and public involvement

All proposals to use data within the SAIL Databank are subject to review by an independent Information Governance Review Panel (IGRP) for privacy risk, data governance and public benefit assessment. The IGRP is made up of a range of independent experts as well as members of the public.

Results

Between 1999 and 2016, 25,523 babies were born alive and affected by congenital anomalies or rare disease of congenital origin, and required a total of 50,705 admissions in infancy. Overall, approximately a quarter of infants had only one admission, over 40% had two or more admissions and more than one third had no

Confidential: For Review Only

admission in infancy. Among infants with isolated anomalies, the largest number of admissions occurred in infants with a cardiovascular anomaly (8% of all admissions of babies with an anomaly).

Table 1 shows that infants who had two or more admissions compared with those who had one or no admission were more likely to live in a deprived area (30.6% versus 28.6% or 26.7%), to have mothers who were younger (33.3% versus 29.3% or 28.2%), nulliparous (45.2% versus 43.6% or 43.3%), and smokers (30.7% versus 27.1% or 25.5%), to be boys (61.2% versus 59.6% or 59.9%), born preterm (21.3% versus 14.0% or 9.1%), to have a low birthweight (21.5% versus 13.5% or 10.1%), or to be one of a multiple pregnancy (3.9% versus 2.9% or 2.4%).

In the univariable analysis, social deprivation, maternal age, parity, maternal smoking, and preterm birth were significantly associated with an increased or decreased risk for repeated admissions for 'all', 'all isolated' and 'all multiple' anomaly subgroups (Table 2). Except for social deprivation and maternal smoking, the same variables (i.e. maternal age and preterm births) which were associated with an increased risk for repeated admissions were also consistently associated with an increased or decreased risk of single admissions of infants for these anomaly subgroups.

Tables 3 and 4 shows the adjusted OR for variables significantly associated with ($p < 0.05$) the number of admissions in infancy by different anomaly or rare disease of congenital origin subgroups. Preterm birth was consistently the strongest risk factor associated with increased 1 and 2+ admissions of infants across all anomalies

Confidential: For Review Only

subgroups with an overall two-and-half fold increased risk of 2+ admissions (aOR: 2.52; 95% CI: 2.25-2.82), and a 68% increased risk of a single admission (aOR: 1.68; 1.47-1.92) compared to term infants. Preterm infants with a cardiovascular anomaly had the highest risk of repeated hospital admissions (aOR: 3.76; 2.86-4.93) compared to term infants with a cardiovascular anomaly. Infant sex was a significant factor for 2+ admissions of infants across all anomaly subgroups. For example, girls with an anomaly had an overall 16% decreased risk of 2+ admissions, compared to boys (aOR: 0.84; 0.78-0.91); and girls with a cardiovascular anomaly had a 25% decreased risk of 2+ admissions, compared to boys (aOR: 0.75; 0.62-0.91). In addition, infants with the most severe and moderately severe CHD had a 2.7 (aOR: 2.72; 1.58-4.71) and 6.3 (aOR: 6.25; 4.47-8.74) folds increased risk of 2+ admissions respectively compared to infants with a less severe CHD. Infants with the most severe CHD had 61% reduced risk of being admitted at all (aOR: 0.39; 0.16-0.96). For infants with multiple anomalies, those whose mothers were aged ≤24 years had a 22% increased risk of 2+ admissions (aOR: 1.22; 1.06-1.41), while those whose mothers were aged ≥ 35 had a 46% decreased risk of 2+ admissions (aOR: 0.54; 0.47-0.63) and a 42% decreased risk of single admissions (aOR: 0.58; 0.48-0.69) compared to those whose mothers were aged 25-29 years. Maternal smoking was associated with an increased risk of 2+ admissions for all anomalies (aOR: 1.20; 1.10-1.31) and all isolated anomalies (aOR: 1.27; 1.13-1.41). The effects of each variable did not vary substantially when adjusted for year of birth (Supplementary Table 2).

Among infants who had at least one admission, maternal age, parity, maternal smoking, and infant sex were not associated with having admissions for causes

Confidential: For Review Only

related to the anomaly as opposed to admissions for other causes (Supplementary Tables 3 and 4). However, preterm infants were less like to have anomaly-specific admissions (aOR: 0.47; 0.42-0.52), whereas infants with mothers having a history of anomalies in previous pregnancies were more likely to have anomaly-specific admissions (aOR: 1.26; 1.11-1.43).

Discussion

Using linked de-identified data from CARIS, PEDW and ONS, we investigated a comprehensive range of risk factors associated with hospital admissions during infancy in babies born with congenital anomalies or rare diseases of congenital origin. Preterm birth was the strongest risk factor associated with an increased risk of one and two or more infant hospitalisation across all anomaly or rare disease of congenital origin subgroups. In addition, infants with two or more admissions had a higher risk profile than those with only one or no admission. Infants who had two or more admissions were more likely to be boys, born preterm, or to have mothers who were younger and smokers or for those who were born with a moderately severe CHD anomaly, compared to those with one or no admission.

Previous studies have shown that infants with a congenital anomaly were associated with a significantly high risk of hospitalisation compared to infants without an anomaly in the general population due to anomaly-related comorbidities and complications, which require ongoing inpatient treatment and investigations [6, 20-22].

Confidential: For Review Only

Maternal and infant factors have an impact on hospitalisation in infant with congenital anomalies through different potential mechanisms. For example, previous studies have shown that teenage mothers may have an increased risk of adverse neonatal outcomes due to inadequate antenatal care and social support, and other behavioural determinants such as maternal smoking, alcohol and recreational drug misuse compared to older mothers [23]. Infants born to older mothers tend to have a reduced risk of unintentional injuries, an increased uptake of routine immunisation in childhood and may receive better parenting [24]. It has been shown that maternal smoking during pregnancy increases the risk of respiratory and infectious diseases in infants [25-27]; possible mechanisms include intrauterine stress and impaired immune system due to nicotine substances and tobacco smoking, although the exact biological mechanism is not clearly defined [28]. In our subgroup analysis, we found no significant difference in the relationship between maternal age and anomaly-specific admissions or other-cause admissions, or those between maternal smoking and anomaly-specific admissions or other-cause admissions, which suggests that the mechanisms by which maternal age and smoking are risk factors for hospitalisation of infants with an anomaly are similar for anomaly-specific admissions and admissions due to other causes.

Preterm birth has consistently been shown to be associated with an increased risk of infant morbidity and hospitalisation; preterm infants generally have a lower physiological reserve and a higher susceptibility to comorbidities and infections compared to term infants [29]. In addition, clinicians may be more cautious and adopt a lower threshold for admitting preterm infants with a congenital anomaly

Confidential: For Review Only

when complications arise [30]. Previous twin studies have shown that boys have an increased risk of respiratory distress syndrome, intrauterine growth restriction and overall morbidities compared with girls and therefore are more likely to require hospitalisation [16, 31-33]. Our subgroup analysis suggests that many infants with congenital anomalies who were born preterm required hospitalisation for conditions related to prematurity rather than the congenital anomaly, although it is possible that clinicians tend to ascribe 'prematurity' as the primary cause of admission for preterm babies regardless of their anomaly status.

Infants born with the most severe CHD (e.g. single ventricle and hypoplastic heart syndromes) often experience life-threatening events at birth and hence the mortality risk among these infants is considerably higher during their first admission compared to infants with less severe CHD [34, 35]. On the other hand, infants born with a moderately severe CHD are now more likely to survive due to advances in diagnostics, and medical and surgical interventions [36]; however, they are also likely to experience long-term morbidities and complications [5, 37, 38], which may explain the highest risk of repeated hospitalisation in this group of infants.

The risk factors for increased hospitalisation in infants with congenital anomalies found in this study are similar to known risk factors for hospitalisation in the general paediatric population, in which previous studies have found that being a boy and maternal smoking are associated with preterm birth leading to increased risk of hospitalisation [39, 40] Having a major anomaly increases the chance of an infant being born preterm [41, 42], and they may contribute to the same chain of event leading to hospitalisation.

Confidential: For Review Only

The main strengths of this study include the national study population, robust study design and data collection. Infants with congenital anomalies or rare diseases of congenital origin were identified from CARIS, an active surveillance and multiple source reporting system covering all livebirths in Wales which maximises case finding and internal validity. The use of routinely collected data from PEDW is particularly advantageous for this hospitalisation study due to the large size of the dataset and long period of data collection. A further strength relates to the broad definition of congenital anomalies, which includes structural and chromosomal defects, as well as rare diseases of congenital origin such as metabolic, endocrine and congenital blood disorders. Consequently, we were able to investigate risk factors associated with hospitalisation of infants with a wide range of congenital anomalies and rare diseases of congenital origin.

There are several limitations. As PEDW is a hospital administrative dataset, data on admissions was not always complete which can be due to infants being treated in a specialist centre outside the catchment area of NHS Wales. In addition, the number of variables explored in this study was restricted by what was available in the dataset. It is possible that institutional factors such as outpatient follow ups which were not available in this dataset are also important determinants of hospitalisation of infants with congenital anomalies.

Conclusions

Infants with congenital anomalies and rare diseases of congenital origin have a high risk of hospitalisation in infancy. Our findings can help clinicians to identify infants

Confidential: For Review Only

who are likely to require hospitalisation according to their socio-demographic and clinical characteristics. Public health interventions such as promoting good prenatal care and smoking cessation may have an important role in reducing the need for hospitalisation in infants with a congenital anomaly.

Confidential: For Review Only

What is known about the subject:

- Congenital anomalies are a leading cause of infant morbidity and hospitalisation.
- Evidence about the risk factors contributing to the hospitalisation of infants with congenital anomalies is limited.

What this study adds

- Infants who had ≥2 admissions were more likely to be boys, born preterm, born with a moderately severe CHD, or have mothers who were younger and smokers, compared to those with ≤1 admission.
- Preterm birth is the strongest risk factor for hospitalisation in infants with congenital anomalies or rare diseases of congenital origin.
- The mechanisms by which maternal age, smoking and infant sex are risk factors for hospitalisation are similar for anomaly-specific admissions and admissions due to other causes.

Confidential: For Review Only

Acknowledgements

We thank the SAIL team for providing the datasets and their assistance with data linkage for this study, and the CARIS team for their support on the dataset.

Funding

This study is part-funded by the National Institute for Health Research (NIHR) Policy Research Programme, conducted through the NIHR Policy Research Unit in Maternal Health and Care, 108/0001. The views expressed are those of the author(s) and not necessarily those of the NIHR or the Department of Health and Social Care.

Peter Ho is funded by a Clarendon – Green Templeton College – Nuffield Department of Population Health Scholarship, University of Oxford.

Competing interests

None.

Confidential: For Review Only

Contributorship statement:

PH contributed to the design of the study, acquisition, analysis, interpretation of data, drafting and revising it critically, approved the final version to be published, and agrees to be accountable for all aspects of the work in ensuring that questions related to the accuracy or integrity of any part of the work are appropriately investigated and resolved.

MQ and JK contributed to the design of the study, acquisition, analysis, and interpretation of data for the work; supervised the project and revised it critically; approved the final version to be published; and agree to be accountable for all aspects of the work in ensuring that questions related to the accuracy or integrity of any part of the work are appropriately investigated and resolved.

DT contributed to the acquisition, interpretation of data for the study, revised it critically, approved the final version to be published, and agrees to be accountable for all aspects of the work in ensuring that questions related to the accuracy or integrity of any part of the work are appropriately investigated and resolved.

For peer review only

Table 1: Factors associated with hospitalisation of infants with any anomaly or rare disease of congenital origin.

Characteristics	Number of admissions		
	2+<br>No. of infants (%)<br>*n = 10,785*	1<br>No. of infants (%)<br>*n = 6,043*	0<br>No. of infants (%)<br>*n = 8,695*
Socio-demographic factors			
Townsend quintile			
1 (least deprived)	1,542 (14.3)	991 (16.4)	1,272 (16.3)
2	1,645 (15.3)	956 (15.8)	1,316 (16.9)
3	1,928 (17.9)	1,102 (18.3)	1,571 (20.1)
4	2,351 (21.8)	1,257 (20.8)	1,558 (20.0)
5 (most deprived)	3,296 (30.6)	1,727 (28.6)	2,080 (26.7)
Maternal ethnicity			
White	6,729 (94.5)	3,547 (93.8)	4,252 (95.0)
Other	394 (5.5)	236 (6.2)	224 (5.0)
Maternal age at birth, years			
Mean (SD) (year)	*27.8 (6.3)*	*28.3 (6.2)*	*28.6 (6.2)*
Median (IQR)	*28 (23-32)*	*28 (24-33)*	*29 (24-33)*
≤ 24	3,596 (33.3)	1,768 (29.3)	2,204 (28.2)
25 – 29	2,880 (26.7)	1,697 (28.1)	2,096 (26.8)
30 – 34	2,585 (24.0)	1,526 (25.3)	2,046 (26.2)
≥ 35	1,720 (16.0)	1,050 (17.4)	1,468 (18.9)
Maternal factors			
Parity			
Nulliparous	4,572 (45.2)	2,454 (43.6)	3,029 (43.3)
≥ 1	5,535 (54.8)	3,173 (56.4)	3,973 (56.7)
Multiple fetus			
Yes	417 (3.9)	175 (2.9)	185 (2.4)
No	10,365 (96.1)	5,866 (97.1)	7,640 (97.6)
Maternal smoking			
Smoker	2,200 (30.7)	1,036 (27.1)	1,136 (25.5)
Non/ Ex smoker	4,958 (69.3)	2,783 (72.9)	3,315 (74.5)
Anomalies in previous pregnancies			
Yes	928 (12.0)	506 (12.2)	582 (12.0)
No	6,811 (88.0)	3,637 (87.8)	4,280 (88.0)
Infant factors			
Infant sex			
Male	6,595 (61.2)	3,599 (59.6)	5,205 (59.9)
Female	4,187 (38.8)	2,441 (40.4)	3,486 (40.1)
Birthweight, grams			
Mean (SD)	*3,024 (810)*	*3,207 (697)*	*3,286 (660)*
Median (IQR)	*3,140 (2,610-3,570)*	*3,280 (2,860-3,660)*	*3,350 (2,950-3,710)*
< 2500 (low birthweight)	2,311 (21.5)	815 (13.5)	806 (10.1)
≥ 2500	8,441 (78.5)	5,209 (86.4)	7,166 (89.9)
Gestational age, week			
Mean (SD)	*37.9 (3.3)*	38.6 (2.6)	39.0 (2.3)
Median (IQR)	*39 (37-40)*	39 (38-40)	39 (38-40)
< 37 (preterm)	2,287 (21.3)	844 (14.0)	727 (9.1)
≥ 37 (term)	8,465 (78.7)	5,178 (86.0)	7,249 (90.9)
Note: Missing data are not shown due to risk of statistical disclosure.			

Confidential: For Review Only

Table 2: Univariable analyses: factors associated with repeated (2+) and single (1) admissions by anomaly/rare disease subgroup.

	2+ admissions			1 admission		
	Unadjusted OR[2]			Unadjusted OR[2]		
Factors	All	Isolated	Multiple	All	Isolated	Multiple
Townsend quintile						
1	1 (reference)	1 (reference)	1 (reference)	1 (reference)	1 (reference)	1 (reference)
2	1.03 (0.93-1.14)	1.04 (0.92-1.18)	1.06 (0.88-1.28)	0.93 (0.83-1.05)	0.88 (0.77-1.01)	1.09 (0.87-1.37)
3	1.01 (0.92-1.12)	1.06 (0.93-1.20)	0.93 (0.78-1.10)	0.90 (0.81-1.01)	0.88 (0.77-1.01)	0.92 (0.74-1.14)
4	1.24 (1.13-1.37)	1.23 (1.08-1.39)	1.24 (1.04-1.47)	1.04 (0.93-1.16)	1.02 (0.89-1.16)	1.08 (0.87-1.34)
5	1.31 (1.19-1.43)	1.32 (1.18-1.48)	1.28 (1.08-1.51)	1.07 (0.96-1.18)	1.02 (0.90-1.15)	1.17 (0.96-1.44)
Maternal ethnicity						
White	1 (reference)	1 (reference)	1 (reference)	1 (reference)	1 (reference)	1 (reference)
Other	1.11 (0.94-1.32)	1.10 (0.88-1.38)	1.06 (0.82-1.39)	1.26 (1.05-1.52)	1.34 (1.06-1.69)	1.13 (0.82-1.56)
Maternal age at delivery, years						
≤ 24	1.19 (1.10-1.28)	1.16 (1.05-1.27)	1.23 (1.07-1.41)	0.99 (0.91-1.09)	0.97 (0.87-1.07)	1.05 (0.88-1.25)
25 – 29	1 (reference)	1 (reference)	1 (reference)	1 (reference)	1 (reference)	1 (reference)
30 – 34	0.92 (0.85-1.00)	0.89 (0.81-0.98)	0.94 (0.81-1.09)	0.92 (0.84-1.01)	0.93 (0.83-1.04)	0.90 (0.75-1.07)
≥ 35	0.53 (0.49-0.58)	0.50 (0.45-0.56)	0.55 (0.48-0.64)	0.55 (0.50-0.61)	0.54 (0.48-0.61)	0.58 (0.48-0.69)
Parity						
Nulliparous	1 (reference)	1 (reference)	1 (reference)	1 (reference)	1 (reference)	1 (reference)
≥ 1	0.73 (0.69-0.77)	0.73 (0.68-0.79)	0.76 (0.68-0.84)	0.78 (0.73-0.84)	0.78 (0.72-0.85)	0.80 (0.71-0.91)
Multiple fetus						
Yes	1.66 (1.39-1.98)	1.92 (1.53-2.39)	1.20 (0.89-1.60)	1.23 (1.00-1.52)	1.43 (1.11-1.84)	0.87 (0.60-1.27)
No	1 (reference)	1 (reference)	1 (reference)	1 (reference)	1 (reference)	1 (reference)
Maternal smoking						
Smoker	1.30 (1.19-1.41)	1.32 (1.19-1.48)	1.24 (1.09-1.43)	1.09 (0.99-1.20)	1.06 (0.94-1.20)	1.14 (0.96-1.34)
Non/Ex smoker	1 (reference)	1 (reference)	1 (reference)	1 (reference)	1 (reference)	1 (reference)
Anomalies in previous pregnancies						
Yes	1.00 (0.89-1.11)	1.04 (0.91-1.20)	0.81 (0.67-0.99)	0.98 (0.86-1.11)	1.00 (0.86-1.16)	0.90 (0.70-1.14)
No	1 (reference)	1 (reference)	1 (reference)	1 (reference)	1 (reference)	1 (reference)
Infant sex[1]						
Male	1 (reference)	1 (reference)	1 (reference)	1 (reference)	1 (reference)	1 (reference)
Female	0.95 (0.90-1.00)	0.94 (0.87-1.01)	0.88 (0.79-0.97)	1.01 (0.95-1.08)	1.07 (0.99-1.16)	0.86 (0.76-0.97)
Gestational age[1], week						
< 37	2.95 (2.70-3.23)	3.25 (2.89-3.64)	2.15 (1.87-2.49)	1.79 (1.61-1.98)	1.91 (1.67-2.18)	1.48 (1.24-1.77)
≥ 37	1 (reference)	1 (reference)	1 (reference)	1 (reference)	1 (reference)	1 (reference)

[1]Variable identified for inclusion a priori. [2]Statistically significant at p < 0.1 for unadjusted OR. Note: OR is relative to the risk in the zero admission patients.

Confidential: For Review Only

Table 3: Multivariable analyses: factors associated with repeated (2+) and single (1) admissions by anomaly or rare disease of congenital origin subgroup.

	2+ admissions			1 admission		
	Adjusted OR[2]			Adjusted OR[2]		
Factors	All	Isolated	Multiple	All	Isolated	Multiple
Maternal age at delivery, years						
≤ 24	1.17 (1.06-1.30)		1.22 (1.06-1.41)	1.00 (0.89-1.12)		1.04 (0.88-1.24)
25 – 29	1 (reference)		1 (reference)	1 (reference)		1 (reference)
30 – 34	0.99 (0.89-1.10)		0.94 (0.81-1.09)	0.94 (0.83-1.06)		0.90 (0.75-1.07)
≥ 35	0.93 (0.82-1.04)		0.54 (0.47-0.63)	0.95 (0.83-1.09)		0.58 (0.48-0.69)
Maternal smoking						
Smoker	1.20 (1.10-1.31)	1.26 (1.13-1.41)		1.06 (0.96-1.17)	1.04 (0.92-1.18)	
Non/Ex smoker	1 (reference)	1 (reference)		1 (reference)	1 (reference)	
Infant sex[1]						
Male	1 (reference)	1 (reference)	1 (reference)	1 (reference)	1 (reference)	1 (reference)
Female	0.84 (0.78-0.91)	0.82 (0.74-0.90)	0.90 (0.81-1.00)	0.95 (0.87-1.04)	1.01 (0.91-1.12)	0.87 (0.77-0.99)
Gestational age[1], week						
< 37	2.52 (2.25-2.83)	2.83 (2.41-3.31)	2.19 (1.90-2.53)	1.68 (1.47-1.92)	1.85 (1.55-2.21)	1.50 (1.26-1.79)
≥ 37	1 (reference)	1 (reference)	1 (reference)	1 (reference)	1 (reference)	1 (reference)

[1]*Variable identified for inclusion a priori.*
[2]*For 'All' anomaly subgroup, adjusted variables include maternal age at delivery, maternal smoking, infant sex and gestational age; for 'isolate' anomaly subgroup, adjusted variables include maternal smoking, infant sex and gestation age; for 'multiple' anomaly subgroup, adjusted variables include maternal age at delivery, infant sex and gestational age. Statistical significance at $p < 0.05$.*

Confidential: For Review Only

Table 4: Unadjusted and adjusted factors associated with repeated (2+) and single (1) admissions of infants with cardiovascular anomalies.

	2+ admissions		1 admission	
	Cardiovascular, *n = 997*			
Factors	Unadjusted OR[2]	Adjusted OR[3]	Unadjusted OR[2]	Adjusted OR[3]
Townsend quintile				
1	1 (reference)		1 (reference)	
2	1.11 (0.80-1.53)		0.80 (0.56-1.12)	
3	1.35 (0.99-1.85)		0.94 (0.68-1.31)	
4	1.10 (0.81-1.50)		1.05 (0.77-1.43)	
5	1.40 (1.05-1.85)		0.97 (0.72-1.30)	
Maternal ethnicity				
White	1 (reference)		1 (reference)	
Other	0.82 (0.49-1.38)		0.91 (0.53-1.57)	
Maternal age at delivery, years				
≤ 24	1.20 (0.95-1.53)		0.94 (0.72-1.23)	
25 – 29	1 (reference)		1 (reference)	
30 – 34	1.02 (0.80-1.31)		1.08 (0.83-1.41)	
≥ 35	0.57 (0.43-0.75)		0.67 (0.51-0.90)	
Parity				
Nulliparous	1 (reference)		1 (reference)	
≥ 1	0.92 (0.76-1.11)		0.86 (0.70-1.05)	
Multiple fetus				
Yes	1.86 (1.15-3.00)		1.55 (0.91-2.62)	
No	1 (reference)		1 (reference)	
Maternal smoking				
Smoker	1.39 (1.07-1.79)		1.07 (0.81-1.42)	
Non/Ex smoker	1 (reference)		1 (reference)	
Anomalies in previous pregnancies				
Yes	1.32 (0.94-1.86)		1.20 (0.84-1.71)	
No	1 (reference)		1 (reference)	
Infant sex[1]				
Male	1 (reference)	1 (reference)	1 (reference)	1 (reference)
Female	0.66 (0.55-0.79)	0.75 (0.62-0.91)	0.95 (0.78-1.15)	0.95 (0.78-1.15)
Gestational age[1], week				
< 37	3.33 (2.55-4.35)	3.76 (2.86-4.93)	1.97 (1.46-2.65)	1.97 (1.46-2.66)
≥ 37	1 (reference)	1 (reference)	1 (reference)	1 (reference)
CHD Disease Severity[1]				
Less severity	1 (reference)	1 (reference)	1 (reference)	1 (reference)
Moderate severity	5.83 (4.19-8.11)	6.25 (4.47-8.74)	1.14 (0.75-1.75)	1.18 (0.77-1.81)
Most severe	2.67 (1.57-4.57)	2.72 (1.58-4.71)	0.38 (0.15-0.94)	0.39 (0.16-0.96)

[1]Variable identified for inclusion a priori. [2]Statistically significant at $p < 0.1$ for the unadjusted OR. [3]Adjusted variables include infant sex, gestational age and disease severity. Statistically significant at $p < 0.05$.

Confidential: For Review Only

References

1. Yoon, P.W., Olney, R.S., Khoury, M.J., Sappenfield, W.M., Chavez, G.F. and Taylor, D., 1997. Contribution of birth defects and genetic diseases to pediatric hospitalizations: a population-based study. *Archives of pediatrics & adolescent medicine*, *151*(11), pp.1096-1103.
2. Cherian, J., Staggers, K.A., Pan, I.W., Lopresti, M., Jea, A. and Lam, S., 2016. Thirty-day outcomes after postnatal myelomeningocele repair: a National Surgical Quality Improvement Program Pediatric database analysis. *Journal of Neurosurgery: Pediatrics*, *18*(4), pp.416-422.
3. Bradnock, T.J., Marven, S., Owen, A., Johnson, P., Kurinczuk, J.J., Spark, P., Draper, E.S. and Knight, M., 2011. Gastroschisis: one year outcomes from national cohort study. *Bmj*, *343*, p.d6749.
4. Derrington, T.M., Kotelchuck, M., Plummer, K., Cabral, H., Lin, A.E., Belanoff, C., Shin, M., Correa, A. and Grosse, S.D., 2013. Racial/ethnic differences in hospital use and cost among a statewide population of children with Down syndrome. *Research in developmental disabilities*, *34*(10), pp.3276-3287.
5. Marston, A.P., Patel, T., Nguyen, S.A. and White, D.R., 2019. Short-Term Risk Factor Profile of Pediatric Choanal Atresia Repair Using ACS-NSQIP National Database. *Annals of Otology, Rhinology & Laryngology*, *128*(9), pp.855-861.
6. Medrano, C., Garcia-Guereta, L., Grueso, J., Insa, B., Ballesteros, F., Casaldaliga, J., Cuenca, V., Escudero, F., de la Calzada, L.G., Luis, M. and Luque, M., 2007. Respiratory infection in congenital cardiac disease. Hospitalizations in young children in Spain during 2004 and 2005: the CIVIC Epidemiologic Study. *Cardiology in the Young*, *17*(4), pp.360-371.
7. Fitzsimons, K.J., Copley, L.P., Deacon, S.A. and van der Meulen, J.H., 2013. Hospital care of children with a cleft in England. *Archives of disease in childhood*, *98*(12), pp.970-974.
8. Razzaghi, H., Dawson, A., Grosse, S.D., Allori, A.C., Kirby, R.S., Olney, R.S., Correia, J. and Cassell, C.H., 2015. Factors associated with high hospital resource use in a population-based study of children with orofacial clefts. *Birth Defects Research Part A: Clinical and Molecular Teratology*, *103*(2), pp.127-143.

Confidential: For Review Only

9. Congenital Anomaly Register and Information Service (CARIS) 2018, *Introduction. Congenital Anomaly Register and information Service*, CARIS, viewed 8 March 2019, <http://www.caris.wales.nhs.uk/introduction>.
10. National Health Service (NHS) Wales Information Service 2020, *PEDW Data Online*, NHS Wales Information Service, viewed 28 December 2019, <http://www.infoandstats.wales.nhs.uk/page.cfm?orgid=869&pid=40977>.
11. Office for National Statistics (ONS) 2015, *Statistical bulletin: Birth Summary Tables, England and Wales: 2014,* ONS, viewed 8 September 2019, <https://www.ons.gov.uk/peoplepopulationandcommunity/birthsdeathsandmarriages/livebirths/bulletins/birthsummarytablesenglandandwales/2015-07-15>.
12. NHS Wales Digital Health and Care Wales, 2021, *NHS Wales Data Dictionary,* NHS Wales Digital Health and Care Wales, viewed 23 May 2021, <http://www.datadictionary.wales.nhs.uk/>.
13. European Surveillance of Congenital Anomalies (EUROCAT) 2013, EUROCAT Guide 1.4: Instruction for the registration of congenital anomalies. EUROCAT Central Registry, University of Ulster. Last update version 28/12/2018, viewed 25 August 2019, <https://eu-rd-platform.jrc.ec.europa.eu/sites/default/files/Full_Guide_1_4_version_28_DEC2018.pdf>.
14. World Health Organization (WHO), 2004. ICD-10: international statistical classification of diseases and related health problems: tenth revision.
15. Butler, A.S. and Behrman, R.E. eds., 2007. *Preterm birth: causes, consequences, and prevention*. National academies press.
16. Stevenson, D.K., Verter, J., Fanaroff, A.A., Oh, W., Ehrenkranz, R.A., Shankaran, S., Donovan, E.F., Wright, L.L., Lemons, J.A., Tyson, J.E. and Korones, S.B., 2000. Sex differences in outcomes of very low birthweight infants: the newborn male disadvantage. *Archives of disease in childhood-fetal and neonatal edition*, *83*(3), pp.F182-F185.
17. Islam, S., Yasui, Y., Kaul, P., Marelli, A.J. and Mackie, A.S., 2016. Congenital heart disease hospitalizations in Canada: a 10-year experience. *Canadian Journal of Cardiology*, *32*(2), pp.197-203.

https://mc.manuscriptcentral.com/bmjpo

18. Mackie, A.S., Tran, D.T., Marelli, A.J. and Kaul, P., 2017. Cost of congenital heart disease hospitalizations in Canada: a population-based study. *Canadian Journal of Cardiology*, *33*(6), pp.792-798.

19. StataCorp. 2013. *Stata Statistical Software: Release 13*. College Station, TX: StataCorp LP.

20. Colvin, L. and Bower, C., 2009. A retrospective population-based study of childhood hospital admissions with record linkage to a birth defects registry. *BMC pediatrics*, *9*(1), p.32.

21. Wynn, J., Krishnan, U., Aspelund, G., Zhang, Y., Duong, J., Stolar, C.J., Hahn, E., Pietsch, J., Chung, D., Moore, D. and Austin, E., 2013. Outcomes of congenital diaphragmatic hernia in the modern era of management. *The Journal of pediatrics*, *163*(1), pp.114-119.

22. Frid, C., Annerén, G., Rasmussen, F., Sundelin, C. and Drott, P., 2002. Utilization of medical care among children with Down's syndrome. *Journal of Intellectual Disability Research*, *46*(4), pp.310-317.

23. Harper, L.M., Chang, J.J. and Macones, G.A., 2011. Adolescent pregnancy and gestational weight gain: do the Institute of Medicine recommendations apply?. *American journal of obstetrics and gynecology*, *205*(2), pp.140-e1.

24. Sutcliffe, A.G., Barnes, J., Belsky, J., Gardiner, J. and Melhuish, E., 2012. The health and development of children born to older mothers in the United Kingdom: observational study using longitudinal cohort data. *Bmj*, *345*, p.e5116.

25. DiFranza, J.R., Aligne, C.A. and Weitzman, M., 2004. Prenatal and postnatal environmental tobacco smoke exposure and children's health. *Pediatrics*, *113*(Supplement 3), pp.1007-1015.

26. Håberg, S.E., Stigum, H., Nystad, W. and Nafstad, P., 2007. Effects of pre-and postnatal exposure to parental smoking on early childhood respiratory health. *American journal of epidemiology*, *166*(6), pp.679-686.

27. Yusuf, H.R., Rochat, R.W., Baughman, W.S., Gargiullo, P.M., Perkins, B.A., Brantley, M.D. and Stephens, D.S., 1999. Maternal cigarette smoking and invasive meningococcal disease: a cohort study among young children in metropolitan Atlanta, 1989-1996. *American journal of public health*, *89*(5), pp.712-717.

https://mc.manuscriptcentral.com/bmjpo

28. Weinsheimer, R.L. and Yanchar, N.L., 2008. Impact of maternal substance abuse and smoking on children with gastroschisis. *Journal of pediatric surgery*, *43*(5), pp.879-883.

29. Björ, O. and Bråbäck, L., 2003. A retrospective population based trend analysis on hospital admissions for lower respiratory illness among Swedish children from 1987 to 2000. *BMC Public Health*, *3*(1), p.22.

30. Mackie, A.S., Ionescu-Ittu, R., Pilote, L., Rahme, E. and Marelli, A.J., 2008. Hospital readmissions in children with congenital heart disease: a population-based study. *American heart journal*, *155*(3), pp.577-584.

31. Lisi, A., Botto, L.D., Rittler, M., Castilla, E., Bianchi, F., Botting, B., De Walle, H., Erickson, J.D., Gatt, M., De Vigan, C. and Irgens, L., 2005. Sex and congenital malformations: an international perspective. *American Journal of Medical Genetics Part A*, *134*(1), pp.49-57.

32. Mulla, Z.D., Plavsic, S.K., Ortiz, M., Nuwayhid, B.S. and Ananth, C.V., 2013. Fetal sex pairing and adverse perinatal outcomes in twin gestations. *Annals of epidemiology*, *23*(1), pp.7-12.

33. Kent, A.L., Wright, I.M. and Abdel-Latif, M.E., 2012. Mortality and adverse neurologic outcomes are greater in preterm male infants. *Pediatrics*, *129*(1), pp.124-131.

34. van Dongen, E.I., Glansdorp, A.G., Mildner, R.J., McCrindle, B.W., Sakopoulos, A.G., VanArsdell, G., Williams, W.G. and Bohn, D., 2003. The influence of perioperative factors on outcomes in children aged less than 18 months after repair of tetralogy of Fallot. *The Journal of thoracic and cardiovascular surgery*, *126*(3), pp.703-710.

35. Desai, J., Aggarwal, S., Lipshultz, S., Agarwal, P., Yigazu, P., Patel, R., Seals, S. and Natarajan, G., 2017. Surgical Interventions in Infants Born Preterm with Congenital Heart Defects: An Analysis of the Kids' Inpatient Database. *The Journal of pediatrics*, *191*, pp.103-109.

36. Holst, K.A., Said, S.M., Nelson, T.J., Cannon, B.C. and Dearani, J.A., 2017. Current interventional and surgical management of congenital heart disease: specific focus on valvular disease and cardiac arrhythmias. *Circulation research*, *120*(6), pp.1027-1044.

37. Smith, A.H., Doyle, T.P., Mettler, B.A., Bichell, D.P. and Gay, J.C., 2015. Identifying predictors of hospital readmission following congenital heart surgery through

Confidential: For Review Only

analysis of a multiinstitutional administrative database. *Congenital heart disease*, *10*(2), pp.142-152.

38. Duppenthaler, A., Ammann, R.A., Gorgievski-Hrisoho, M., Pfammatter, J.P. and Aebi, C., 2004. Low incidence of respiratory syncytial virus hospitalisations in haemodynamically significant congenital heart disease. *Archives of disease in childhood*, *89*(10), pp.961-965.
39. Stock, S.J. and Bauld, L., 2020. Maternal smoking and preterm birth: An unresolved health challenge. *PLoS Medicine*, *17*(9), p.e1003386.
40. Zeitlin, J., Saurel-Cubizolles, M.J., De Mouzon, J., Rivera, L., Ancel, P.Y., Blondel, B. and Kaminski, M., 2002. Fetal sex and preterm birth: are males at greater risk?. *Human reproduction*, *17*(10), pp.2762-2768.
41. Butler, A.S. and Behrman, R.E. eds., 2007. *Preterm birth: causes, consequences, and prevention*. National academies press.
42. Dolan, S.M., Gross, S.J., Merkatz, I.R., Faber, V., Sullivan, L.M., Malone, F.D., Porter, T.F., Nyberg, D.A., Comstock, C.H., Hankins, G.D. and Eddleman, K., 2007. The contribution of birth defects to preterm birth and low birth weight. *Obstetrics & Gynecology*, *110*(2), pp.318-324.

Confidential: For Review Only

Supplementary Table 1: Inclusion of anomaly and rare disease of congenital origin subgroups.

Subgroup	Classification of anomaly and rare disease of congenital origin included
All anomalies; all isolated anomalies; and all multiple anomalies	• Nervous system;<br>• Eye, ear, face and neck;<br>• Cardiovascular system;<br>• Respiratory and clefts;<br>• Abdominal wall defects and diaphragmatic hernia;<br>• Upper and lower gastrointestinal;<br>• Genitourinary;<br>• Limb and skeletal;<br>• Blood disorders;<br>• Endocrine and Metabolic;<br>• Neoplasm;<br>• Syndromes and congenital malformation syndromes;<br>• Maternal Infection leading to malformations.
Cardiovascular anomalies	• Congenital heart defects and diseases of the circulatory system.

Confidential: For Review Only

Supplementary Table 2: Year of birth added as a covariate in the final model as a sensitivity analysis.

	2+ admissions				1 admission			
	Adjusted OR				Adjusted OR			
Factors	All	Isolated	Multiple	Cardiovascular	All	Isolated	Multiple	Cardiovascular
Maternal age at delivery, years								
≤ 24	1.18 (1.06-1.30)		1.22 (1.06-1.41)		1.00 (0.89-1.12)		1.04 (0.88-1.24)	
25 – 29	1 (reference)		1 (reference)		1 (reference)		1 (reference)	
30 – 34	0.99 (0.89-1.10)		0.94 (0.81-1.09)		0.94 (0.83-1.06)		0.90 (0.75-1.07)	
≥ 35	0.93 (0.82-1.04)		0.54 (0.47-0.63)		0.95 (0.84-1.09)		0.58 (0.48-0.69)	
Maternal smoking								
Smoker	1.20 (1.10-1.31)	1.26 (1.13-1.41)			1.06 (0.96-1.17)	1.04 (0.92-1.18)		
Non/Ex smoker	1 (reference)	1 (reference)			1 (reference)	1 (reference)		
Infant sex[1]								
Male	1 (reference)	1 (reference)	1 (reference)	1 (reference)	1 (reference)	1 (reference)	1 (reference)	1 (reference)
Female	0.84 (0.78-0.91)	0.82 (0.74-0.90)	0.90 (0.81-1.00)	0.75 (0.62-0.91)	0.95 (0.87-1.04)	1.01 (0.91-1.12)	0.87 (0.77-0.99)	0.95 (0.78-1.15)
Gestational age[1], week								
< 37	2.52 (2.25-2.83)	2.83 (2.41-3.31)	2.19 (1.90-2.53)	3.74 (2.84-4.91)	1.68 (1.47-1.92)	1.85 (1.55-2.21)	1.50 (1.26-1.79)	1.97 (1.45-2.64)
≥ 37	1 (reference)	1 (reference)	1 (reference)	1 (reference)	1 (reference)	1 (reference)	1 (reference)	1 (reference)
CHD Disease Severity[1]								
Less severity	N/A	N/A	N/A	1 (reference)	N/A	N/A	N/A	1 (reference)
Moderate severity				6.31 (4.51-8.83)				1.20 (0.78-1.84)
Most severe				2.76 (1.60-4.77)				0.39 (0.16-0.98)
For each year increment between 1999 and 2016.	1.00 (0.99-1.01)	1.00 (0.99-1.01)	1.00 (0.99-1.01)	0.99 (0.97-1.01)	0.99 (0.98-1.00)	0.99 (0.97-1.00)	1.00 (0.98-1.01)	0.98 (0.96-1.00)

[1]*Variable identified for inclusion a priori. N/A = not applicable.*

Confidential: For Review Only

Supplementary Table 3: Factors associated with cause-specific admissions among infants with any anomaly or rare disease of congenital origin.

	Anomaly-specific admissions, no. of infants (%)	Other-cause admissions, no. of infants (%)	Unadjusted OR (95% CI)	P-value[1]
Socio-demographic factors				
Townsend quintile				
1 (least deprived)	772 (15.4)	1,709 (14.9)	1 (reference)	0.253
2	767 (15.3)	1,780 (15.6)	0.95 (0.85-1.08)	
3	932 (18.6)	2,042 (17.9)	1.01 (0.90-1.13)	
4	1,093 (21.8)	2,432 (21.3)	0.99 (0.89-1.11)	
5 (most deprived)	1,439 (28.8)	3,472 (30.4)	0.92 (0.83-1.02)	
Maternal ethnicity				
White	3,101 (94.2)	6,948 (94.2)	1 (reference)	0.978
Other	191 (5.8)	429 (5.8)	1.00 (0.84-1.19)	
Maternal age at birth, years *(n = 29,400)*				
≤ 24	1,570 (31.3)	3,680 (32.1)	0.97 (0.89-1.06)	0.712
25 – 29	1,363 (27.1)	3,103 (27.1)	1 (reference)	
30 – 34	1,251 (24.9)	2,782 (24.3)	1.02 (0.93-1.22)	
≥ 35	831 (16.6)	1,891 (16.5)	1.00 (0.90-1.11)	
Maternal factors				
Parity				
Nulliparous	2,101 (41.9)	4,782 (41.7)	1 (reference)	0.856
≥ 1	2,914 (58.1)	6,674 (58.3)	0.99 (0.93-1.06)	
Multiple fetus				
Yes	153 (3.1)	427 (3.7)	0.81 (0.67-0.98)	0.028
No	4,862 (96.9)	11,029 (96.3)	1 (reference)	
Maternal smoking				
Smoker	946 (28.0)	2,218 (30.1)	0.90 (0.83-0.99)	0.027
Non/ Ex smoker	2,430 (72.0)	5,147 (69.9)	1 (reference)	
Anomalies in previous pregnancies				
Yes	374 (10.4)	1,028 (12.8)	1.27 (1.12-1.44)	<0.001
No	3,231 (89.6)	6,981 (87.2)	1 (reference)	
Infant factors				
Infant sex				
Male	3,013 (60.1)	6,964 (60.8)	1 (reference)	0.405
Female	1,999 (39.9)	4,489 (39.2)	1.03 (0.96-1.10)	
Birthweight, grams				
< 2500	598 (11.9)	2,448 (21.4)	0.50 (0.45-0.55)	<0.0001
≥ 2500	4,417 (88.0)	9,008 (78.6)	1 (reference)	
Gestational age, week				
$< 37^{+0}$	577 (11.5)	2,470 (21.6)	0.47 (0.43-0.52)	<0.0001
$\geq 37^{+0}$	4,438 (88.5)	8,986 (78.4)	1 (reference)	

[1]P < 0.1 was considered as statistically significant. Missing data are not shown due to risk of statistical disclosure.

Confidential: For Review Only

Supplementary Table 4: Factors associated with cause-specific admissions among infants with any anomaly or rare disease of congenital origin: multivariable analysis.

Risk factors	Crude OR (95% CI)	P-value[1]	aOR[2] (95% CI)	P-value[3]
Anomalies in previous pregnancies				
Yes	1.27 (1.12-1.44)	<0.001	1.26 (1.11-1.43)	<0.001
No	1 (reference)		1 (reference)	
Gestational age, week				
< 37 (preterm)	0.47 (0.43-0.52)	<0.0001	0.47 (0.42-0.52)	<0.0001
≥ 37 (term)	1 (reference)		1 (reference)	

[1]P-value of the univariable analysis < 0.1 was considered as statistically significant. [2]Mutually adjusted odds ratios for other factors in the table. [3]P-value of the multivariable analysis < 0.05 was considered as statistically significant.

Confidential: For Review Only
